# Institutional Priority-Setting for Novel Drugs and Therapeutics: A Qualitative Systematic Review

**DOI:** 10.34172/ijhpm.2024.7494

**Published:** 2024-02-10

**Authors:** Daniel E. Wang, Maram Hassanein, Yasmeen Razvi, Randi Zlotnik Shaul, Avram Denburg

**Affiliations:** ^1^Department of Paediatrics, University of Toronto, Toronto, ON, Canada.; ^2^Department of Bioethics, The Hospital for Sick Children, Toronto, ON, Canada.; ^3^Temerty Faculty of Medicine, University of Toronto, Toronto, ON, Canada.; ^4^Child Health Evaluative Sciences, SickKids Research Institute, Toronto, ON, Canada.; ^5^Division of Paediatric Haematology/Oncology, The Hospital for Sick Children, Toronto, ON, Canada.

**Keywords:** Health Technology Assessment, Paediatrics, Rare/Orphan Diseases, Institutional Policy, High-Cost Drugs, Resource Allocation

## Abstract

**Background:** There is a lack of guidance on approaches to formulary management and funding for high-cost drugs and therapeutics by individual healthcare institutions. The objective of this review was to assess institutional approaches to resource allocation for such therapeutics, with a particular focus on paediatric and rare disease populations.

**Methods:** A search of Embase and MEDLINE was conducted for studies relevant to decision-making for off-formulary, high-cost drugs and therapeutics. Abstracts were evaluated for inclusion based on the Simple Multiple-Attribute Rating Techniques (SMART) criteria. A framework of 30 topics across 4 categories was used to guide data extraction and was based on findings from the initial abstract review and previous health technology assessment (HTA) publications. Reflexive thematic analysis was conducted using QSR NVivo 12 software.

**Results:** A total of 168 studies were included for analysis. Only 4 (2%) focused on paediatrics, while 21 (12%) centred on adults and the remainder (85%) did not specify. Thirty-two (19%) studies discussed the importance of high-cost therapeutics and 34 (23%) focused on rare/orphan drugs. Five themes were identified as being relevant to institutional decision-making for high-cost therapeutics: institutional strategy, substantive criteria, procedural considerations, guiding principles and frameworks, and operational activities. Each of these themes encompassed several sub-themes and was complemented by a sixth category specific to paediatrics and rare diseases.

**Conclusion:** The rising cost of novel drugs and therapeutics underscores the need for robust, evidence-based, and ethically defensible decision-making processes for health technology funding, particularly at the hospital level. Our study highlights practices and themes to aid decision-makers in thinking critically about institutional, substantive, procedural, and operational considerations in support of legitimate decisions about institutional funding of high-cost drugs and therapeutics, as well as opportunities and challenges that exist for paediatric and rare disease populations.

## Background

 The rising cost of drugs and therapeutics in health systems internationally has created difficult decisions about which drugs to fund and for whom in the face of limited public sector resources.^1,2^ Institutions at varying levels of the healthcare system, from governments to individual hospitals, are increasingly challenged to identify novel interventions that are both beneficial to patients and cost-effective. This poses a greater challenge for vulnerable populations, such as children, the elderly,and persons with rare or orphan diseases, where access to funding may be more limited than for traditionally well-supported populations and disease areas.^3-6^

 In response to this challenge, many institutions and healthcare leaders are turning to formal processes and decision tools to aid funding decisions. Established processes for health technology assessment (HTA), incorporating a range of frameworks and tools, are increasingly integrated into public health system approaches to resource allocation for health technologies, including drugs and therapeutics.^4,7-9^ For decision-makers at all levels of the healthcare system, a robust decision-making process with articulated principles, goals and criteria can help manage the complexity of funding decisions surrounding high-cost, innovative therapies.^10-13^ While no formal, standardized threshold exists to distinguish between high- and low-cost drugs, definitions of high-cost therapeutics have included those which are expensive, generate excessive costs, and represent a disproportionate cost relative to the total cost of the agent in terms of volume and duration.^14,15^ Specific thresholds often vary between institutions, with the authors’ institution defining high-cost therapies as those costing more than CAD$ 50 000 annually or more than CAD$ 5000 per administration. It should be noted that this value was established several years ago and the numerical threshold used to define expensive therapeutics will understandably change over time, in keeping with dynamic economies.

 Formulary management pertains to the continuous evaluation and updating of an approved list of medications for use by a healthcare organization, which is a process supported by best available evidence and clinical expertise, and is typically the responsibility of institutional drug and therapeutics committees.^16^ A dearth of guidance for funding of high-cost drugs and therapeutics persists concerning formulary management by healthcare delivery institutions — notably, academic hospitals — where many of the ultimate decisions are made regarding formulary inclusion and patient access to novel therapies. The lack of such formal processes at individual hospitals — where formulary management decisions directly impact patient access to medicines — remains a widespread challenge. Some healthcare delivery institutions have published experiences with the development and implementation of accountability pathways or procedural approaches to formulary management that are grounded on institutional values such as transparency, accountability, and consistency.^11,17-19^ However, there remains a lack of published evidence on priority-setting processes for funding drugs and therapeutics at the hospital level.

 There is a particular need for formal governance approaches to such decision-making as it pertains to drugs for rare diseases and/or paediatric populations, for which standard approaches to HTA are often insufficient or absent altogether. Scant literature exists to address the unique considerations relevant to drug priority-setting and formulary management in paediatrics or rare diseases. These considerations include, among others: (1) *ethical* questions about priority populations and the role of patient and public values in approaches to resource allocation, (2) *substantive* questions about the nature and strength of evidence available to inform funding decisions, and (3) *procedural* questions about which perspectives to include in resource allocation decisions and how best to publicize and revisit those decisions.

 To help fill these gaps in knowledge, we conducted a systematic review of the extant literature on approaches to resource allocation for high-cost drugs, with a particular focus on paediatric and rare diseases indications, to guide evidence-informed approaches to hospital funding decisions, specifically formulary management, for high-cost drugs and therapeutics.

## Methods

 The following research question guided our review: how do institutions approach decision-making for off-formulary high-cost drugs and therapeutics and are there special considerations made for rare diseases or paediatric populations? The present work applies primarily to the private and public not-for-profit space, with a specific focus on patient-facing healthcare institutions, where the costs associated with therapeutics are borne by the institution rather than out-of-pocket by patients. While hospitals were the principal focus of our work, our search strategy was deliberately inclusive in regards to the types of healthcare institutions to ensure that all relevant data was included. We undertook a qualitative systematic literature review following a critical interpretive synthesis approach,which draws upon both qualitative research inquiry as well as systematic review principles to synthesize diverse forms of evidence.^20^

 We performed a broad search strategy of Embase and MEDLINE to capture English language studies from 2000 to 2020 relevant to decision-making for off-formulary, high-cost therapeutics. The list of Medical Subject Headings (MESH) terms used is in Supplementary file 1. Given that the development of innovative, high-cost agents is rapidly evolving, both with respect to their increasingly prohibitive costs and the rarity of conditions they address, our sample was later restricted to literature published in the last 5 years to maximize the relevance of the sample to current debates on decision-making for novel drugs and therapeutics. We evaluated abstracts per the Simple Multiple-Attribute Rating Techniques (SMART), using 3 a priori inclusion criteria (description of the decision-making process; organizational or institutional decision-making; and description of values in decision-making) and 3 a priori exclusion criteria (government policy-making; laboratory or bench-based scientific research; and cost-effectiveness analysis, clinical guideline development or clinical trial results).^21^ We assessed each abstract using a 3-point Likert scale (0 for not relevant, 3 for relevant) for each inclusion criterion up to a maximum 9-point score (Supplementary file 2). All team members scored a sub-sample of abstracts to ensure consensus. Two team members (DEW and MH) independently evaluated all relevant abstracts, including categorization of those abstracts according to the age of the population, level of health system scope, cost of therapeutic intervention, and scope of illness or disease area, with discrepancies resolved through discussion within the research team. The kappa measure of agreement was calculated between the 2 reviewers. Abstracts that scored 8 or higher underwent full-text review.

 After removing studies with insufficient data (eg, conference presentations or abstracts without full papers), both reviewers (DEW and MH) independently extracted data from included studies. A detailed framework was developed to guide extraction from eligible studies (Supplementary file 3), which consisted of 30 topics across 4 high-level categories. This was based on findings from the initial abstract review and prior HTA publications. In particular, these 4 categories reflected a grouping of decision criteria incorporated into most HTA approaches: (1) substantive considerations that include information or data content that will be used to inform the decision, (2) procedural considerations that include the steps or process that will be used to make the decision, (3) prioritization considerations that include methods or mechanisms by which different criteria will be evaluated to facilitate decision-making, and (4) technology or tool considerations that include technical aids and templates that will be used to standardize submissions and reporting.

 Extracted data was uploaded to QSR NVivo 12 software for qualitative coding and reflexive thematic analysis, as outlined by Braun and Clarke, using a constant comparative analysis approach.^22–24^ One reviewer (MH) performed initial coding and development of preliminary themes, which were further developed by a second reviewer (DEW) and ultimately refined by the research team.

## Results

**Figure 1:**
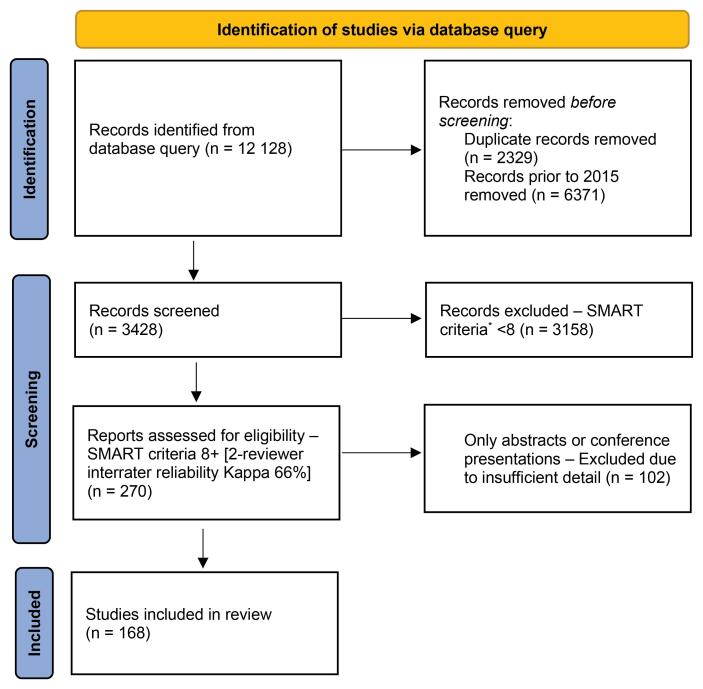
PRISMA Flow Diagram of the Literature Review Process. Abbreviation: PRISMA, Preferred Reporting Items for Systematic Reviews and Meta-Analyses. ^*^SMART criteria to score the most relevant abstracts for analysis: (a) description of decision-making process; (b) focus on organizational or institutional decision-making; and (c) description of values in decision-making. Abbreviation: SMART, Simple Multiple-Attribute Rating Techniques.

 Our initial literature review yielded 12 128 total records. We removed 2239 duplicates, leaving 9799 unique records. Narrowing the results to the most recent 5 years resulted in 3428 relevant abstracts. Applying SMART inclusion and exclusion criteria, we categorized abstracts with a score of ≥8 (out of 9 possible points) (n = 270). Removing abstracts lacking full-text manuscripts resulted in 168 studies for detailed analysis. The Preferred Reporting Items for Systematic Reviews and Meta-Analyses (PRISMA) flow diagram for study selection is shown in Figure 1.

 Our detailed review of 168 studies yielded 4 (2%) focused on the paediatric population, 21 (12%) focused on adults, and the remainder (85%) did not specify the population of interest (Table, Panel A). Seven (4%) of the studies were focused at the hospital level, whereas a large majority of studies (74%) were focused at the national or international level (Table, Panel B). Only 32 studies (19%) mentioned specifically the importance of high-cost therapeutics (Table, Panel C). Thirty-nine (23%) of the studies focused on cancer therapies and 34 (20%) were focused on rare or orphan drugs. Other focus areas included autoimmune or rheumatologic conditions, metabolic diseases, and medical technology/devices, in addition to general studies that had no specific illness focus (Table, Panel D). A full summary of included studies can be found in Supplementary file 4.

**Table:** Breakdown of Studies by Areas of Research Relevance

**A. Studies by Age of Patient Population**	**No.**	**%**
Paediatrics	4	2
Adult	21	13
Not specified	143	85
Grand total	168	100
**B. Studies by Healthcare System Level**	**No.**	**%**
Hospital	7	4
Local/regional	7	4
National	77	46
Hospital, regional and national	1	1
International	48	29
Not specified	28	17
Grand total	168	100
**C. Studies by Cost of Therapeutic Intervention**	**No.**	**%**
Specific high-cost interventions	32	19
Not high-cost intervention	103	61
Not specified	33	20
Grand total	168	100
**D. Studies by Illness or Disease Area**	**No.**	**%**
Not specified	61	36
Oncology	39	22
Rare/orphan drugs	34	20
Medical technology/devices	3	2
Metabolic	2	1
Rheumatologic/autoimmune	2	1
Experimental drugs	2	1
Other specified	25	15
Grand total	168	100

**Figure 2:**
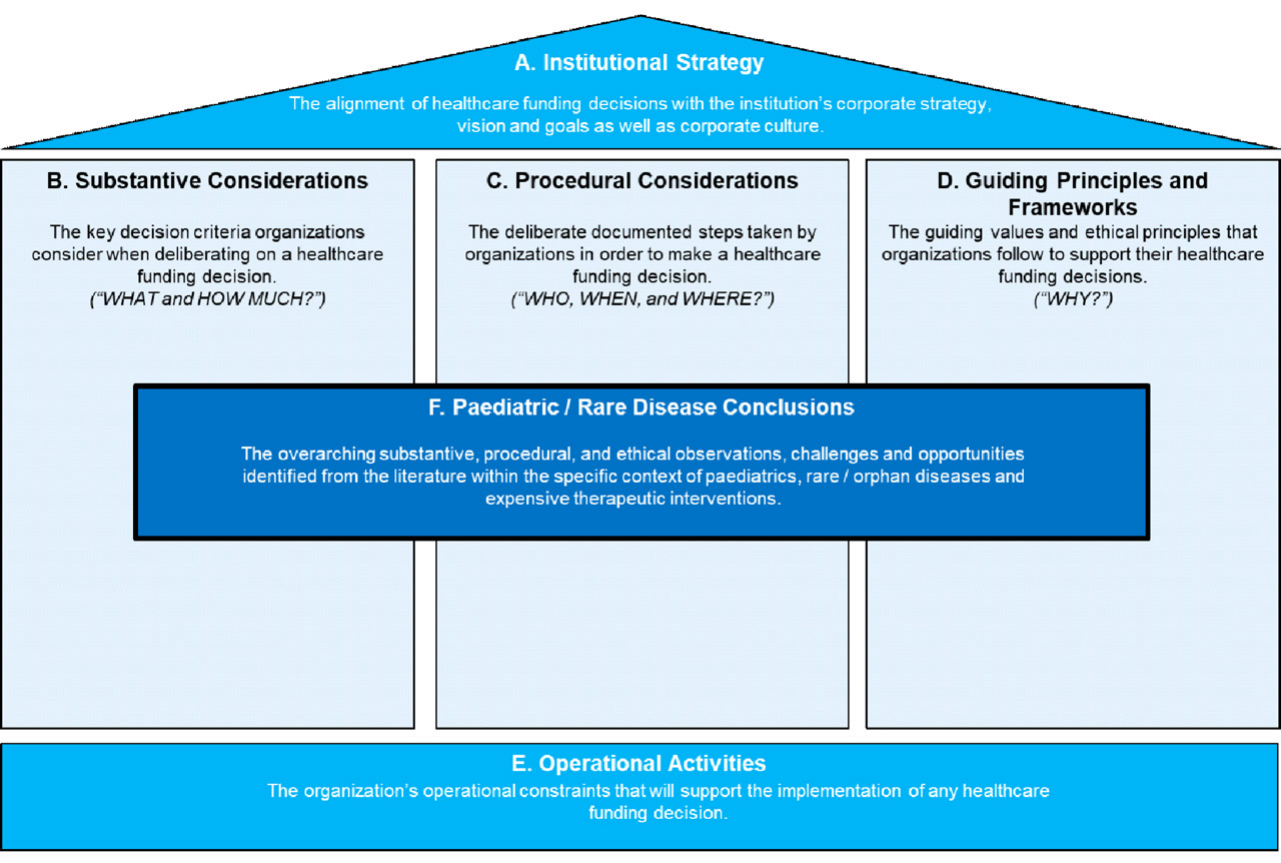
Thematic Framework.

 Our findings yield 5 foundational categories relevant to institutional decision-making on novel and high-cost drugs and therapeutics: institutional strategy, substantive criteria, procedural criteria, guiding principles and frameworks, and operational activities. These were complemented by a sixth category specific to paediatrics and rare/orphan diseases (Figure 2).

**Figure 3:**
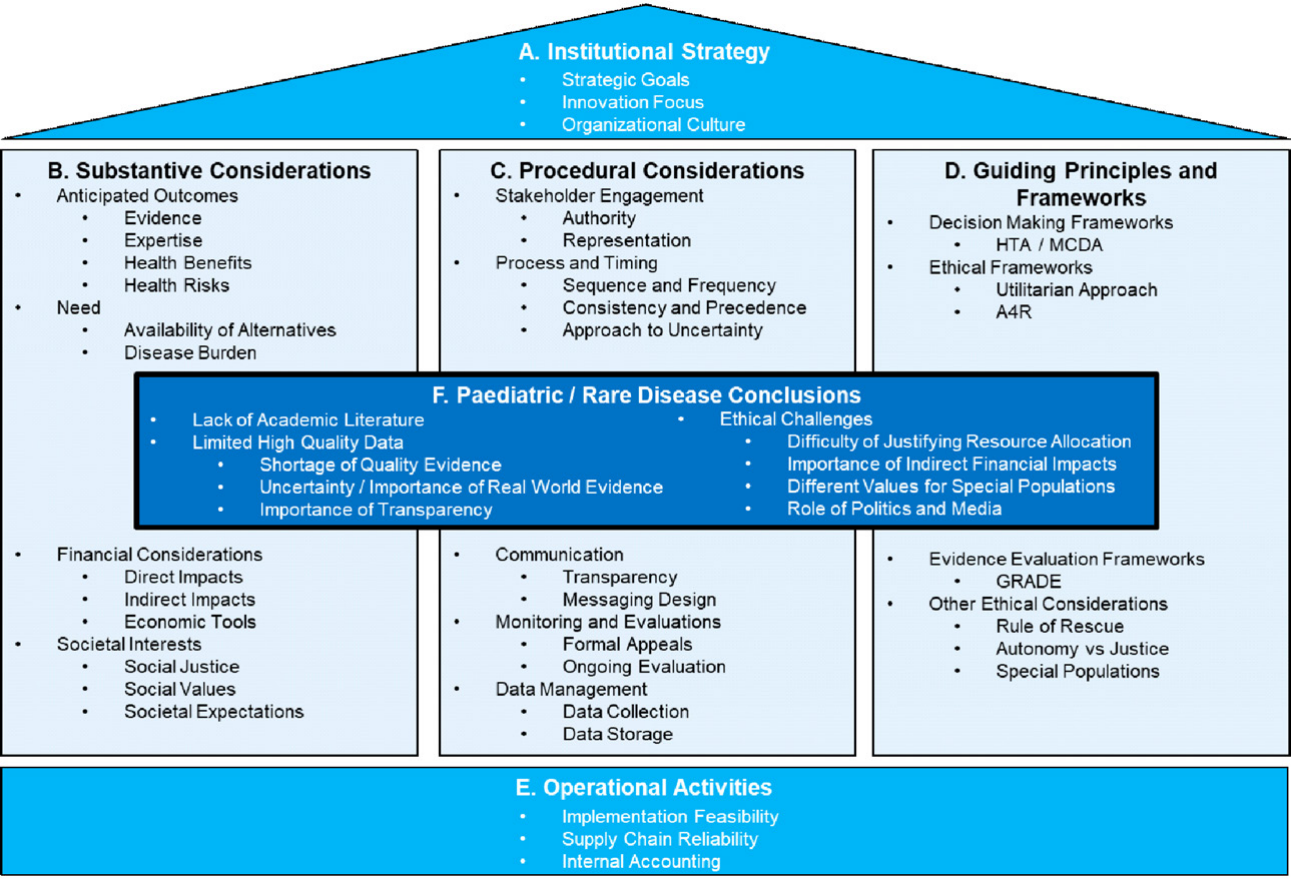
Thematic Subjects and Topics. Abbreviations: MCDA, multi-criteria decision analysis; HTA, health technology assessment.

 Each category subsumes several key themes. *Institutional strategy* identifies themes of alignment of healthcare funding decisions with the institution’s corporate strategy, vision, goals and culture. *Substantive considerations* identify themes pertaining to the key decision criteria institutions consider when determining healthcare funding priorities. *Procedural considerations* identify themes pertaining to the deliberate steps taken by institutions to make healthcare funding decisions. *Guiding principles and frameworks* identify themes pertaining to the guiding values and ethical principles institutions follow to support their healthcare funding decisions. *Operational activities* identify themes pertaining to the institution’s human resource, logistics, and accounting structures that support the implementation of healthcare funding decisions. *Paediatric /rare disease conclusions* identify themes inferred from the 5 preceding categories, specific to decision-making about high-cost innovative therapies in the context of a paediatric academic healthcare centre. The identified themes — grouped by category into 19 discrete subjects covering 36 unique topics — highlight predominant trends and recurrent challenges relevant to healthcare institutional decision-making on funding for high-cost innovative therapies (Figure 3, Supplementary file 5).

###  A. Institutional Strategy

 Available evidence suggests that decision-makers working within a given institutional context were more likely to align their healthcare funding decisions with the institution’s corporate strategy, vision, and goals, as well as consider the corporate culture around transparency and shared decision-making.^25,26^ Funding decisions were often made with the institution’s broader strategic goals in mind, including institutional financial realities and objectives, local and regional health metrics, health system priorities, and core institutional values — especially with respect to equity, diversity, and inclusion. Additionally, decision-makers at all levels of the healthcare system tried to promote scientific innovation through healthcare funding decisions, even when such innovation and its benefits were difficult to quantify or evaluate at the decision time point.^26^

###  B. Substantive Criteria

 Institutions typically considered a set of core criteria in technology funding decisions, including anticipated outcomes, patient and population health needs, and financial costs. Alongside these, broader societal values and expectations have come to figure more prominently in institutional decision-making over time. The assessment of anticipated health outcomes was usually based on a combination of scientific evidence, clinical expertise, and known health risks of the proposed intervention. Real-world data was increasingly proposed and used to supplement data from clinical trials. Determination of need for a given healthcare intervention was informed by the burden and severity of the disease and the availability of alternative treatments at the time of the funding decision. Economic impacts were frequently a key consideration, usually measured through cost-effectiveness and budget impact analyses. Corollary impacts such as opportunity costs to institutions and indirect costs to patient families (eg, lost economic productivity, transportation, and accommodation costs) were also identified as salient considerations to institutional decision-making. We note recent examples where decision-makers have attempted to incorporate social values and/or societal expectations into healthcare funding decisions. Prominent examples include: the Swedish government endorsing the use of “human dignity” as a relevant ethical principle in health resource allocation, which has led to positive drug funding decisions in the face of higher cost-efficiency ratios; UK’s National Institute for Health and Care Excellence affording “special priority” for disadvantaged populations for HTA funding decisions; and a range of authors and institutions espousing the importance of equity of access in allocative decision-making.^27-32^

###  C. Procedural Criteria

 Institutions often used a formal sequence of activities by which funding decisions were made to ensure consistency in the funding decision-making process. These activities included stakeholder engagement, data collection, data analysis and deliberation, and decision communication. Which and how many stakeholders to include in decision-making processes were core issues deliberated upon in the available literature, with many institutions endorsing the inclusion of a wide range of stakeholders but also placing particular emphasis on the involvement of relevant medical authorities, including doctors and representatives from regulatory bodies. Having a documented process with formal timelines also promoted consistency in the process but did not guarantee a consistent decision outcome. Indeed, divergence in funding recommendations on a given technology was acknowledged as an acceptable outcome of fair deliberative processes.^33^ When communicating the results of a funding decision, most stakeholders emphasized the importance of transparency, with a clearly communicated rationale or process description as critical to legitimate decision-making. Decision-makers also monitored changes in scientific data to re-evaluate prior decisions. Using data templates and databases to streamline information collection and storage was also common, regardless of institutional size.

###  D. Guiding Principles and Frameworks

 Institutions often followed specific or well-established decision principles and ethical frameworks to guide their decision-making. Prominent examples include multiple criteria decision analysis (MCDA) techniques and the Accountability for Reasonableness (A4R) framework. MCDA techniques attempt to evaluate multiple, often competing, priorities among stakeholders to establish a unified outcome.^21^ The A4R framework provides an ethics-informed approach to promote a fair decision-making process based on 5 conditions: relevance, publicity, revision and appeals, empowerment, and enforcement.^11,17,18^ Having an established process or set of guiding principles helped stakeholders identify and understand implicit value judgements. Using a peer-reviewed system, such as the GRADE (Grading of Recommendations, Assessment, Development, and Evaluations) framework, to evaluate the quality of scientific evidence helped achieve a common understanding between stakeholders.^34^ Other common ethical considerations included incorporating the rule of rescue, highlighting the role of justice, and attempting to address vulnerable or other special populations in decision-making.

###  E. Operational Activities

 Similar to institutional strategy, health technology funding decisions were often made in the context of considerations related to the feasibility of implementation by the institution. Interventions whose implementation was supported by the institution’s available human resources capacities, supply chain logistics, and internal accounting/cost structures tended to fare better in terms of positive funding decisions.

###  F. Paediatric /Rare Disease Conclusions

 Based on our review, the considerations and determinants that inform paediatric and rare disease funding decisions are not well documented in the academic literature. Evidence on health technology decision-making at the level of individual hospitals, or focused specifically on high-cost therapeutics, is likewise scant.

 When decision-making processes were reported for these populations, they often lacked high-quality scientific evidence. This appeared to be due to challenges, both logistical and ethical, associated with the conduct of clinical trials in paediatric or rare disease populations.^7,35^ Some decision-makers emphasized real-world evidence (RWE) to support their decisions on technology funding in these domains in light of limited trial-based data. However, this notion also raised challenges in the collection and evaluation of real-world data when there are limited peer-reviewed tools for quality appraisal of non-trial data, especially compared to those that are well established for scientific evidence, such as GRADE.^36^ Recognizing the challenges with limited high-quality evidence, decision-makers also placed more importance on process considerations, such as transparency, as legitimators of funding decisions for health technologies in the paediatric and rare disease space.

 Paediatric and rare disease patients were sometimes identified as vulnerable populations and prioritized for positive funding decisions accordingly. However, decision-makers who attempted to redress social imbalances through funding decisions faced challenges in the context of decision-making processes involving a range of stakeholders with varied priorities, such as prioritization of funding for diseases with greater prevalence or population-level burden.^7,37^ A range of stakeholders, however, recognized that funding decisions for paediatric populations, in particular, should consider the indirect impacts of healthcare interventions on caregivers and families. Recent literature also highlighted the role of social media and the broader political landscape as influences over healthcare funding for vulnerable or special populations.^38,39^

## Discussion

 Our analysis shows certain themes are consistent across approaches to healthcare funding decisions for high-cost drugs and therapeutics, while other themes relate uniquely to specific populations, such as paediatric and rare diseases. Themes falling into the first 5 categories identified in Figure 2 seemed to persist, and retain relevance, across a wide array of patient groups, diseases, and health technologies. These include (1) institutional considerations such as strategic goals or attempts to promote innovation, (2) substantive criteria such as healthcare needs, evidence and outcomes, side effects, and direct financial costs, (3) procedural considerations such as broad stakeholder engagement, promotion of transparency, and well-documented processes, (4) the use of formal frameworks to structure decision processes such as MCDA or A4R, and (5) operational considerations such as the feasibility of implementing funding decisions.

 While these themes predominated, we noted several novel or emergent findings. For example, one substantive consideration reported on multiple occasions was the inclusion of social values considerations, in varied ways and to varying degrees, in healthcare decision-making. Correspondingly, a key challenge identified was how such social values considerations, which are often difficult to quantify or render comparable to other relevant forms of evidence, can be meaningfully incorporated into decision-making. A 2020 survey of a national sample of Canadian adults sought to evaluate public preferences related to healthcare resource allocation across a range of settings, which found a consistent preference by participants to allocate scare resources to the paediatric population.^40^ Other guiding principles included equal treatment, relieving suffering, and prioritizing those at greatest risk of mortality, demonstrating the potential of public engagement in priority-setting processes.

 Another notable finding was a relative lack of explicit reference to or clear articulation of, ethical principles as decision-making determinants — particularly in light of an increased scholarly focus on ethics in healthcare decision-making in recent years.^41-43^ Explicit reference to ethical frameworks was rare. Instead, there were many examples where ethical principles were implicitly incorporated across all stages of decision-making. For example, the prioritization of substantive criteria such as societal interests, acknowledgement of the importance of procedural transparency and consistency, and recognition of the value of broad stakeholder consultation are examples of how decision-makers’ implicit values played a significant role in decision-making. This finding suggests an opportunity to improve the rigour and transparency with which ethical reasoning is incorporated and acknowledged in funding decisions for high-cost drugs and therapeutics. Similar concerns are described with respect to more established HTA processes, which have been noted to rarely or only superficially address the role of ethical principles.^44,45^ Ethics working groups have been established to develop ethical frameworks within HTA, and have demonstrated the need for methodological approaches that are context-sensitive and question-based to address ethical issues as they arise.^46^

 Importantly, our review also highlighted the significant resources — notably institutional human resources, governance structures, supporting tools, and technologies — that are required to follow robust healthcare decision-making processes. The administrative burden and associated time to coordinate such activities at an institutional level often resulted in delayed decisions, including in some instances multi-year processes required to arrive at a funding decision. This represents a significant challenge for institutions looking to implement decisions with short turn-around times. One of the barriers to quick funding decisions was the imperative for comprehensive stakeholder engagement, as a substantial amount of time was needed to reach and engage diverse stakeholders as well as to adapt communications to audiences of varying levels of education and healthcare knowledge.

 Perhaps most striking from our review are findings related to drug funding decisions for paediatrics and rare diseases. There was a significant lack of scholarly publications focused on healthcare decision-making for these populations (See Table). While we might anticipate that these populations represent a smaller portion of reported literature compared to adult conditions and/or more prevalent diseases, our analysis confirmed an almost total lack of focused literature therein. A 2021 review by Denburg et al assessed the literature on the normative dimensions of social and health-related policy specific to paediatrics more broadly (n = 123), which revealed 3 central themes of this work: (1) potential, (2) rights, and (3) risks.^47^ These themes were accompanied by a meta-theme recognizing the unique position of children in the family unit and society, which in turn influences the direction of social policies. While these themes have been noted across policy domains, future work should detail the impact and variance of these values across contexts and investigate tensions therein. Our work similarly notes the importance of further evaluating the values specific to children given the current dearth of literature on this topic. Since the completion of this review, Pucchio and Reider have published the results of a cross-sectional survey of all 19 chairs of Canadian paediatrics departments regarding funding and accessibility of high-cost drugs, which revealed that there is inconsistency in funding processes, sources, and overall frustration with the present state of affairs, reflecting a general consensus that funding structures are not meeting the needs of Canadian children.^48^

###  Identified Challenges

 Our review also identified some important challenges when making healthcare funding decisions, whether for specialized populations or not. Many of these relate to ethical considerations – partly because the ethical principles are not well defined a priori, but partly because the challenges themselves are ethical dilemmas without easy solutions. For example, authors highlighted the challenge of attempting to incorporate societal considerations in funding decisions but often found it difficult to do so in the face of diverse and often divergent views about healthcare funding priorities from patient and public stakeholders. Another recurrent ethical and epistemic challenge was how to compare data that was readily quantifiable in dollar terms, such as drug prices or cost-effectiveness ratios, with qualitative data, such as patient testimonies, or novel forms of evidence or elements of value, such as indirect socioeconomic benefits, or the value of innovation. Decision-makers seemed to want to incorporate qualitative data more routinely and rigorously as part of the decision process, but without clear parameters by which to do so, which tended to devalue and de-prioritize qualitative sources of data in favour of quantifiable metrics for decision-making.

 Decision-makers also struggled to deal with uncertainty, especially when funding decisions related to specialized populations such as paediatrics or rare diseases, where rigorous safety and effectiveness data for relevant technologies is often lacking. While some decision processes attempted to use formal decision-modelling techniques such as sensitivity analyses to account for this uncertainty, others did not address uncertainty at all. More recently, authors have started envisioning and promoting RWE and patient-reported outcome measures as rigorous means of supplementing limited clinical trials data on the safety and efficacy of novel drugs and therapeutics for rare indications – either identifying and collecting already-existing RWE during the decision-making process or collecting RWE retrospectively as part of ongoing evaluations.^49,50^

 The final area of challenge relates to the desire to incorporate a breadth of stakeholder perspectives in the decision-making process. The implementation of broad stakeholder engagement, an increasingly accepted practice in healthcare funding decision-making — especially in publicly funded healthcare systems — has created multiple challenges for decision-makers. These challenges included delays in decision-making due to the time required to engage a range of stakeholders; difficulties in having to alter or translate healthcare data into language and information that would be understandable by a variety of audiences; challenges in connecting to and engaging marginalized populations; concerns that stakeholders with vested interests unfairly affected funding decisions; and challenges related to how to reconcile a range of competing stakeholder views.

###  Identified Recommendations 

 Notwithstanding the identified challenges to making evidence-based and ethically sound healthcare funding decisions, the authors also identified useful recommendations to guide decision-makers. These include the importance of incorporating novel forms and sets of data, including RWE as well as patient and public input, when trial-based data is lacking, especially for rare or paediatric populations; establishing a documented decision-making process that allows stakeholders to understand how decisions were made, even if they do not agree with the substance of the decision; using frameworks or checklists to guide the development of funding decisions or policies that relate to healthcare funding; and the importance of ongoing tracking and evaluation of data regarding past decisions to inform future decision-making, as well as the potential for disinvestment. The latter is vital to ensuring that new data, technology, and actual health outcomes are considered as part of the implementation of any funding decision.^51^

###  Implications for Future Policy and Research 

 Our research provides an overview of the key components of healthcare funding decision processes for novel and high-cost drugs and therapeutics, as identified through a rigorous review of the literature. While decision processes are becoming more robust through the use of validated methods, ethical frameworks, and the inclusion of economic evaluation, there is still a paucity of published literature documenting well-established mechanisms to incorporate relevant emerging forms of evidence such as societal values, indirect economic considerations, RWE of effectiveness, and patient-reported outcomes. There is also a lack of reported best practices for addressing the myriad of ethical challenges when dealing with divergent stakeholder views and a lack of rigorous safety and effectiveness data in specialized populations, such as paediatric and rare disease populations. While a number of the themes and challenges identified in our study are core to decision-making for health technologies and recapitulated across the academic literature surveyed, substantial gaps remain in documenting and understanding the distinct challenges and opportunities attached to decision-making for paediatric and rare disease therapies.

 Our study was subject to a few important limitations. Our research applies primarily to both private and public not-for-profit, patient-facing institutions, which may limit its applicability to for-profit settings. Our review of the literature was also limited to English-language journals over the most recent 5-year period; it did not include studies before 2015 or written in other languages. It also did not incorporate a grey literature review of policy documents from individual HTA or healthcare institutions that might have shed additional light on institutional approaches to decision-making for high-cost drugs and therapeutics, whether for paediatric or more general populations. A review of grey literature and individual institutional reports enhanced by qualitative interviews with key healthcare funding decisions would help fill key gaps in understanding optimal decision-making practices for paediatric and rare disease therapies, especially for institutions looking to model their decision-making on established or effective processes in such specialized populations. We plan to undertake such work in future.

## Conclusion

 The mounting cost of novel drugs and therapeutics amidst funding constraints in many international health systems underscores the need for robust, evidence-based, and ethically defensible decision-making processes for health technology funding — not only for formal HTA bodies but for hospitals and patient-facing healthcare institutions more broadly. Our review identifies a widespread need for more transparent and systematic decision-making frameworks at healthcare institutions that make both the inherent ethical challenges and relevant criteria for such decisions more explicit. We also identify a particular need for additional evidence to support funding decision-making in paediatric and rare disease populations that account for the distinct scientific and social values considerations relevant to these groups. Our study sheds light on important recurrent practices and themes that can help decision-makers think carefully about institutional, substantive, procedural, and operational considerations in support of legitimate decisions about institutional funding of high-cost drugs and therapeutics, as well as unique opportunities and challenges that exist for specialized populations such as paediatric and rare disease populations.

## Ethical issues

 Not applicable.

## Competing interests

 Authors declare that they have no competing interests.

## Supplementary files


Supplementary file 1. Database Search Strategy.

Supplementary file 2. SMART Criteria.

Supplementary file 3. Initial Framework for Detailed Literature Analysis.

Supplementary file 4. List of Included Studies for Detailed Analysis.

Supplementary file 5. Summary of Identified Themes, Conclusions and Challenges From Literature Analysis.
