# Supplementary material for: Institutional Priority-Setting for Novel Drugs and Therapeutics: A Qualitative Systematic Review
Source: Int J Health Policy Manag. 2024 Feb 10;13:7494. doi: 10.34172/ijhpm.2024.7494 (PMC11016276; doi:10.34172/ijhpm.2024.7494)
Supplement: Supplementary file 1 — Database Search Strategy. [file ijhpm-13-7494-s001.pdf]

Article title: Institutional Priority-Setting for Novel Drugs and Therapeutics: A Qualitative Systematic Review

Journal name: International Journal of Health Policy and Management (IJHPM)

Authors' information: Daniel E. Wang[1], Maram Hassanein[2], Yasmeen Razvi[3,4], Randi Zlotnik Shaul[1,2,4], Avram Denburg[1,4,5]*

[1]Department of Paediatrics, University of Toronto, Toronto, ON, Canada.

[2]Department of Bioethics, The Hospital for Sick Children, Toronto, ON, Canada.

[3]Temerty Faculty of Medicine, University of Toronto, Toronto, ON, Canada.

[4]Child Health Evaluative Sciences, SickKids Research Institute, Toronto, ON, Canada.

[5]Division of Paediatric Haematology/Oncology, The Hospital for Sick Children, Toronto, ON, Canada.

***Correspondence to:** Avram Denburg; Email: avram.denburg@sickkids.ca

Citation: Wang DE, Hassanein M, Razvi Y, RZ Shaul, Denburg A. Institutional priority-setting for novel drugs and therapeutics: a qualitative systematic review. Int J Health Policy Manag. 2024;13:7494. doi:10.34172/ijhpm.2024.7494

Supplementary file 1. Database Search Strategy

**Database [Platform]** *Searches run April 16, 2020*	**Results**
MEDLINE(R) and Epub Ahead of Print, In-Process & Other Non-Indexed Citations and Daily 1946 [OVID] to April 14, 2020	3361
Embase Classic+Embase [OVID] 1947 to 2020 Week 15	8767
**TOTAL**	**12,128**

Ovid MEDLINE(R) and Epub Ahead of Print, In-Process & Other Non-Indexed Citations and Daily 1946 to April 14, 2020
Search Strategy:

#	Searches	Results
1	Drugs, Investigational/	5629
2	Drug Therapy/ec [Economics]	1216
3	Drug Prescriptions/ec [Economics]	2898
4	Drug Costs/	15920
5	formularies as topic/ or formularies, hospital as topic/	2828
6	Neoplasms/dt [Drug Therapy]	68042
7	Pharmaceutical Preparations/ec [Economics]	1629
8	(drug? adj3 (cancer? or oncolog* or biologic? or rare condition? or rare disease?)).tw,kf.	31825
9	(drug? adj3 (expensive or novel or new or non-formular* or nonformular* or orphan*)).tw,kf.	93186
10	(pharma* adj3 (cancer? or oncolog* or biologic? or rare condition? or rare disease?)).tw,kf.	4316
11	(pharma* adj3 (expensive or novel or new or non-formular* or nonformular* or orphan*)).tw,kf.	19163
12	(chemotherap* adj3 (cost* or expensive or novel or new or non-formular* or nonformular* or ophan*)).tw,kf.	8002

13	Resource Allocation/	8287
14	(allocat* adj3 resource?).tw,kf.	17620
15	or/1-14	256971
16	Decision-making/	93705
17	Decision-making, Organizational/	11087
18	(decision? adj2 (maker? or making)).tw,kf.	153734
19	((decide? or decision?) adj5 (framework? or guidance or gude* or process* or tool? or tree?)).tw,kf.	47704
20	(fund* adj5 (arrang* or decide? or decision? or determin* or guidance or guide?)).tw,kf.	6386
21	or/16-20	239735
22	Cost-Benefit Analysis/	80101
23	"Cost Control"/	21467
24	Drug Costs/	15920
25	Economics, Pharmaceutical/	2922
26	Economics, Medical/	9060
27	Ethics/	9731
28	Evidence-Based Medicine/	72312
29	economics.fs.	419289
30	ethics.fs.	70659
31	Health Priorities/	10699
32	Health Services Accessibility/	72950
33	Social Justice/	12002
34	Social Responsibility/	19218
35	Technology Assessment, Biomedical/	9905
36	(access* adj3 (drug? or treatment?)).tw,kf.	12314
37	(accountabilit* or ethic* or moral* or priorit* or "obligations to society" or social accountabilit* or social justice or social good or social obligation? or social responsibilit* or value?).tw,kf.	2114147
38	(cost? adj3 (allocation? or contain* or control* or minimiz*)).tw,kf.	20071
39	(cost benefit or cost effectiveness or cost utility analys#s or "costs and benefits" or "benefits and costs" or economic evaluation? or marginal analys#s).tw,kf.	81155
40	(economic* adj3 (drug? or pharmacotherap* or medical or healthcare or health care or health service? or hospital?)).tw,kf.	12472
41	evidence-based.tw,kf.	113326

42	(health technolog* assessment? or biomedical technolog* assessment?).tw,kf.	4990
43	or/22-42	2805483
44	15 and 21 and 43	4967
45	limit 44 to yr="2000-current"	3530
46	limit 45 to english language	3361

Embase Classic+Embase 1947 to 2020 Week 15
Search Strategy:

#	Searches	Results
1	new drug/	39214
2	drug costs/	73612
3	(drug? adj3 (cancer? or oncolog* or biologic? or rare condition? or rare disease?)).tw,kw.	46937
4	(drug? adj3 (expensive or novel or new or non-formular* or nonformular* or orphan*)).tw,kw.	135357
5	(pharma* adj3 (cancer? or oncolog* or biologic? or rare condition? or rare disease?)).tw,kw.	7126
6	(pharma* adj3 (expensive or novel or new or non-formular* or nonformular* or orphan*)).tw,kw.	27653
7	(chemotherap* adj3 (costly or expensive or novel or new or non-formular* or nonformular* or ophan*)).tw,kw.	10560
8	resource allocation/	20338
9	(allocat* adj3 resource?).tw,kw.	21978
10	or/1-9	341796
11	Decision-making/	229634
12	clinical decision-making/	44451
13	medical decision-making/	85312
14	(decision? adj2 (maker? or making)).tw,kw.	210169
15	((decid* or decision?) adj5 (framework? or guidance or gude* or process* or tool? or tree?)).tw,kw.	67795
16	(fund* adj5 (arrang* or decid* or decision? or determin* or guidance or guide?)).tw,kw.	8104
17	or/11-16	474764
18	"cost benefit analysis"/	84030
19	"cost control"/	67704
20	"drug cost"/	76787
21	economic evaluation/	15531
22	"cost utility analysis"/	9525
23	pharmacoeconomics/	7267

24	health economics/	37472
25	evidence based medicine/	109168
26	health care planning/	100729
27	health care delivery/	174521
28	social justice/	10253
29	social responsibility/	1230
30	biomedical technology assessment/	14349
31	(access* adj3 (drug? or treatment?)).tw,kw.	18760
32	(accountabilit* or ethic* or moral* or priorit* or "obligations to society" or social accountabilit* or social justice or social good or social obligation? or social responsibilit* or value?).tw,kw.	2947967
33	(cost benefit or cost effectiveness or cost utility analys#s or "costs and benefits" or "benefits and costs" or economic evaluation? or marginal analys#s).tw,kw.	116506
34	(economic* adj3 (drug? or pharmacotherap* or medical or healthcare or health care or health service? or hospital?)).tw,kw.	15392
35	evidence-based.tw,kw.	150840
36	(health technolog* assessment? or biomedical technolog* assessment?).tw,kw.	7048
37	or/18-36	3663943
38	10 and 17 and 37	10718
39	limit 38 to yr=2000-current	9150
40	limit 39 to english	8767
