# Supplementary material for: Institutional Priority-Setting for Novel Drugs and Therapeutics: A Qualitative Systematic Review
Source: Int J Health Policy Manag. 2024 Feb 10;13:7494. doi: 10.34172/ijhpm.2024.7494 (PMC11016276; doi:10.34172/ijhpm.2024.7494)
Supplement: Supplementary file 2 — SMART Criteria. [file ijhpm-13-7494-s002.pdf]

Article title: Institutional Priority-Setting for Novel Drugs and Therapeutics: A Qualitative Systematic Review

Journal name: International Journal of Health Policy and Management (IJHPM)

Authors' information: Daniel E. Wang[1], Maram Hassanein[2], Yasmeen Razvi[3,4], Randi Zlotnik Shaul[1,2,4], Avram Denburg[1,4,5]*

[1]Department of Paediatrics, University of Toronto, Toronto, ON, Canada.

[2]Department of Bioethics, The Hospital for Sick Children, Toronto, ON, Canada.

[3]Temerty Faculty of Medicine, University of Toronto, Toronto, ON, Canada.

[4]Child Health Evaluative Sciences, SickKids Research Institute, Toronto, ON, Canada.

[5]Division of Paediatric Haematology/Oncology, The Hospital for Sick Children, Toronto, ON, Canada.

***Correspondence to:** Avram Denburg; Email: avram.denburg@sickkids.ca

Citation: Wang DE, Hassanein M, Razvi Y, RZ Shaul, Denburg A. Institutional priority-setting for novel drugs and therapeutics: a qualitative systematic review. Int J Health Policy Manag. 2024;13:7494. doi:10.34172/ijhpm.2024.7494

Supplementary file 2. SMART Criteria

Each abstract was reviewed for the following three criteria:

A. **Description of decision-making process**: Does the abstract include an explanation of the process used to determine how decisions were made based on different resource allocation criteria, including if possible, an explicit process for prioritizing different decision criteria?

B. **Focus on hospital decision-making**: Does the abstract focus on decision-making regarding clinical treatments, drugs, therapeutics, or technologies that are applicable at the hospital level?

C. **Description of values in decision-making**: Does the abstract include an explanation of what value drivers are used to make policy / treatment decision with a preferable emphasis on social values, ethics, and fairness in addition to purely clinical, medical or economic analyses?

Each criterion was assessed according to a 3-point scale:

- Yes = 3
- Probably = 2
- Doubtful = 1
- No = 0

The total score for each abstract was the simple addition of the score from each criterion. A maximum score was 9 points per abstract (the sum of 3 points if answered "yes" to each of criteria A, B, and C) and a minimum score was 0 points (the sum of 0 points if answered "no" to each of the criteria A, B, and C)
