# Supplementary material for: Institutional Priority-Setting for Novel Drugs and Therapeutics: A Qualitative Systematic Review
Source: Int J Health Policy Manag. 2024 Feb 10;13:7494. doi: 10.34172/ijhpm.2024.7494 (PMC11016276; doi:10.34172/ijhpm.2024.7494)
Supplement: Supplementary file 3 — Initial Framework for Detailed Literature Analysis. [file ijhpm-13-7494-s003.pdf]

Article title: Institutional Priority-Setting for Novel Drugs and Therapeutics: A Qualitative Systematic Review

Journal name: International Journal of Health Policy and Management (IJHPM)

Authors' information: Daniel E. Wang[1], Maram Hassanein[2], Yasmeen Razvi[3,4], Randi Zlotnik Shaul[1,2,4], Avram Denburg[1,4,5]*

[1]Department of Paediatrics, University of Toronto, Toronto, ON, Canada.

[2]Department of Bioethics, The Hospital for Sick Children, Toronto, ON, Canada.

[3]Temerty Faculty of Medicine, University of Toronto, Toronto, ON, Canada.

[4]Child Health Evaluative Sciences, SickKids Research Institute, Toronto, ON, Canada.

[5]Division of Paediatric Haematology/Oncology, The Hospital for Sick Children, Toronto, ON, Canada.

***Correspondence to:** Avram Denburg; Email: avram.denburg@sickkids.ca

Citation: Wang DE, Hassanein M, Razvi Y, RZ Shaul, Denburg A. Institutional priority-setting for novel drugs and therapeutics: a qualitative systematic review. Int J Health Policy Manag. 2024;13:7494. doi:10.34172/ijhpm.2024.7494

Supplementary file 3. Initial Framework for Detailed Literature Analysis

Category ID	Category	Definition	Key Questions to Consider
**S**	**Substantive criteria**	The substantive or information content that will be used to inform the decision	What information is needed to make this decision?
**P**	**Procedural criteria**	The procedure or process that will be used to make the decision	How will the decision be made? Who needs to be involved? When will it be made and how often?
**R**	**Prioritization methods**	The methods or mechanisms by which different substantive and / or procedural criteria will be evaluated and / or prioritized to facilitate decision-making	How will we decide the relative importance of different criteria?
**T**	**Technology and tools**	The tools and templates that will be used to standardize submissions for evaluation and / or store information including the decision itself	What technical tools or templates should be used to standardize information collection and storage?

Topic ID	Topic	Definition	Key Questions to Consider
**S1**	**Evidence**	The scientific (quantitative or qualitative) data to support the proposed therapeutic intervention	What evidence was used to support the decision? What was the level of such evidence?
**S2**	**Expertise**	The individual expertise, qualifications, and identification of subject matter experts involved in the decision	What subject expertise were involved in the decision? What were their qualifications?
**S3**	**Alternatives/Availability**	Any proposed alternative interventions to the proposed intervention including no intervention	Did the decision consider alternate treatments?
**S4**	**Health Benefits**	The potential benefit of the proposed therapeutic intervention including direct / indirect benefits to patient, family, society	What benefits are described? Are they short or long-term? Are they only health specific?

**S5**	**Health Risks**	The risks / potential harms of the proposed intervention including direct / indirect risks to patient, family, society	What are the risks associated with the treatment? What is the safety profile?
**S6**	**Financial Considerations (Direct and indirect costs)**	The estimated financial cost / benefit to the funding institution of the proposed intervention for the duration of the funder's intervention. Any indirect costs / benefits assessed including opportunity costs to funder, costs / savings to patient / family after the funder's funding duration is complete, downstream health system economic costs	What are the direct costs of the intervention? Are those costs one-time or recurring? Are there any direct financial benefits to this treatment (e.g. cheaper than the current treatment)? Are there any indirect costs associated with this treatment - e.g. opportunity costs, additional health system costs, additional family costs, post-paediatric costs borne by another provider? Are there any indirect benefits of this intervention e.g. health system savings, research benefits for novel drugs, institutional branding opportunities
**S7**	**Disease burden**	Any description of how disease burden is assessed	What are the indicators used for disease burden e.g. financial cost, morbidity or mortality
**S8**	**Health related need**	The need for treatment - what will be the effect on the patient's medical health and quality of life without treatment	How is the patient's need for treatment measured? Any quantifiable criteria?
**S9**	**Economic tools**	Any description of economic tools or analysis used	Is there any description of value or cost-effectiveness, e.g. Incremental Cost Effectiveness Ratio, Budget Impact Analysis or other metrics?
**S10**	**Social Values**	Description or exploration of local / regional norms, patient or family values or cultural or religious beliefs	Are there any specific values, norms, morals that are prevalent in society and influence the decision?
**S11**	**Societal Expectations**	Local society or community rules, laws, regulations, political movements, media expectations including risks and opportunities	Are there any important laws, regulations, political movements that is influencing the decision process or decision itself?
**S12**	**Social Justice**	Explicit mention of accessibility, socioeconomic determinants, equity / diversity / inclusion criteria, or other social determinants of health	Is there any consideration for addressing inequity or social determinants of health as part of this decision?
**S13**	**Other Substantive Criteria**	Other criteria not fitting into the topics above	
**P1**	**Timing and Frequency**	The expected turnaround time for a decision and/ or how often decisions should be made	When should the decision be made? Should these decisions take place at regular intervals?
**P2**	**Consistency / Precedence**	Any discussion of prior decisions influencing future decisions	Does past precedent determine future precedent? Is there any effective statute of limitations if so? Is there any specific discussion of consistency in decision-making process?
**P3**	**Representation**	Identification of stakeholders included in the decision including internal and external to the institution; any patients / families included	Who is involved in the decision? Are they internal or external to the institution? Are there any patients / families involved in the decision? Are there certain experts involved? Are decision-makers representative of important stakeholder groups?
**P4**	**Authority**	Any identification of decision hierarchy or explicit mention of who / what part of the institution ultimately makes the decision	What is the highest level of authority involved in the decision? Does authority matter or is consensus preferred if multiple people are involved in the decision?
**P5**	**Communication**	How decisions are communicated and to whom, including any differences in positive or negative decisions	Do communications differ if the decision if yes or no? How often are stakeholders updated on the decision-making process and decision, if at all?

**P6**	**Transparency**	How the decision-making process ensures transparency, throughout the process and / or regarding the final decision	Is there a clear audit trail of how the decision was made? Are there any efforts to make the decision or decision-making process clear to those not involved? Is information shared with the public?
**P7**	**Evaluation**	How the decision is monitored for effectiveness and / or real-life outcomes	What processes are in place to assess the outcomes (short or long, direct or indirect) of the decision? How do decision-makers determine successful decisions? Do decision-makers follow the patients after treatment and if so, for how long?
**P8**	**Appeals**	Any process / rationale for appealing decisions	What, if any, is the process or rule by which a decision can be appealed?
**P9**	**Miscellaneous**	Other topics not included above	
**R1**	**Ethical Frameworks**	Any explicit mention of ethical frameworks or principles used to inform the decision or prioritize different criteria	Are there any explicit ethical principles mentioned in the article as the basis for decision-making? How are conflicting criteria or principles prioritized?
**R2**	**Multi-Criteria Decision Tools**	Any tools or quantitative techniques to help prioritize different decision-making criteria	What, if any, quantitative or algorithmic methods are used to aid the prioritization process?
**R3**	**Corporate Tools**	Any use of institutional / corporate tools or techniques for decision-making	Do decision-makers use any business decision-making tools to aid the decision such as balanced scorecards, risk matrices, etc.
**R4**	**Other Prioritization Frameworks**	Other prioritization methodologies not covered in the topics above	
**T1**	**Submission Templates**	Description of any forms, templates or method of structuring the information used to evaluate the therapeutic / drug	Is information standardized in any way through forms / templates / submission tools?
**T2**	**Data Storage**	Explanation of how data / information relating to the decision is stored, either internally or elsewhere, and who has access to such information	Where is the decision-making information / application stored? Who has access to such information?
**T3**	**Communication Channels**	The methods by which information is shared among stakeholders	What communication channels (e.g. email, social media, marketing, internal meetings, newsletters, town halls, etc.) are used to disseminate the result of the decision?
**T4**	**Other Tools / Technology**	Other tools or technology used in the decision-making process not covered by the topics above	
