# Supplementary material for: Institutional Priority-Setting for Novel Drugs and Therapeutics: A Qualitative Systematic Review
Source: Int J Health Policy Manag. 2024 Feb 10;13:7494. doi: 10.34172/ijhpm.2024.7494 (PMC11016276; doi:10.34172/ijhpm.2024.7494)
Supplement: Supplementary file 4 — List of Included Studies for Detailed Analysis. [file ijhpm-13-7494-s004.pdf]

Article title: Institutional Priority-Setting for Novel Drugs and Therapeutics: A Qualitative Systematic Review

Journal name: International Journal of Health Policy and Management (IJHPM)

Authors' information: Daniel E. Wang[1], Maram Hassanein[2], Yasmeen Razvi[3,4], Randi Zlotnik Shaul[1,2,4], Avram Denburg[1,4,5]*

[1]Department of Paediatrics, University of Toronto, Toronto, ON, Canada.

[2]Department of Bioethics, The Hospital for Sick Children, Toronto, ON, Canada.

[3]Temerty Faculty of Medicine, University of Toronto, Toronto, ON, Canada.

[4]Child Health Evaluative Sciences, SickKids Research Institute, Toronto, ON, Canada.

[5]Division of Paediatric Haematology/Oncology, The Hospital for Sick Children, Toronto, ON, Canada.

***Correspondence to:** Avram Denburg; Email: avram.denburg@sickkids.ca

Citation: Wang DE, Hassanein M, Razvi Y, RZ Shaul, Denburg A. Institutional priority-setting for novel drugs and therapeutics: a qualitative systematic review. Int J Health Policy Manag. 2024;13:7494. doi:10.34172/ijhpm.2024.7494

Supplementary file 4. List of Included Studies for Detailed Analysis

Title:	Author:	Date:
Setting Healthcare Priorities at the Macro and Meso Levels: A Framework for Evaluation	Barasa EWM, Sassy; English, Mike; Cleary, Susan	2015
Novel oral anticoagulants: Recommendations for patient evaluation, treatment initiation, follow-up and perioperative management	Baumann SH, A.; El-Battrawy, I.; Renker, M.; Akin, I.	2015
A practical guide for using registry data to inform decisions about the cost effectiveness of new cancer drugs: lessons learned from the PHAROS registry	Blommestein HMF, Margreet G.; Uyl-de Groot, Carin A.	2015
Governance of conditional reimbursement practices in The Netherlands	Boon WM, Luis; Koopmanschap, Marc	2015
Improving Cross-Sector Comparisons: Going Beyond the Health-Related QALY	Brazier JT, Aki	2015
Improving the efficiency and quality of the value assessment process for companion diagnostic tests: The companion test assessment tool (CAT)	Canestaro WJP, D. E.; Garrison, L. P.; Dubois, R.; Veenstra, D. L.	2015
Withdrawal of anticancer therapy in advanced disease: a systematic literature review	Clarke GJ, S.; Corrie, P.; Kuhn, I.; Barclay, S.	2015
'Real-world' health care priority setting using explicit decision criteria: a systematic review of the literature	Cromwell IP, Stuart J.; Mitton, Craig	2015
Why orphan drug coverage reimbursement decision-making needs patient and public involvement	Douglas CMWW, Elizabeth; Burgess, Michael; Lynd, Larry D.	2015
Making decisions about medications in critically ill children: A survey of canadian pediatric critical care clinicians	Duffett MC, K.; Vanniyasingam, T.; Thabane, L.; Cook, D. J.	2015

TRANSDISCIPLINARITY IN STRATEGIC DECISIONS FOR ONCOLOGICAL TREATMENTS	Fernandes IR, M. C.; Portela, S.	2015
IS THE LINK BETWEEN HEALTH AND WEALTH CONSIDERED IN DECISION MAKING? RESULTS FROM A QUALITATIVE STUDY	Garau MS, Koonal Kirit; Sharma, Priya; Towse, Adrian	2015
Societal preferences for rheumatoid arthritis treatments: Evidence from a discrete choice experiment	Harrison MM, C.; Shojania, K.; Bansback, N.	2015
Developing a patient-directed policy framework for managing orphan and ultra-orphan drugs throughout their lifecycle	Menon DS, Tania; Dunn, Andrea; Wong-Rieger, Durhane	2015
Reimbursed price of orphan drugs: A value based framework	Mincarone PL, C. G.; Sabina, S.; Tordrup, D.; Taruscio, D.; Kanavos, P.	2015
From market access to patient access: overview of evidence-based approaches for the reimbursement and pricing of pharmaceuticals in 36 European countries	Panteli DE, Helene; Nolting, Alexandra; Busse, Reinhard; Kulig, Michael	2015
Value-based reimbursement decisions for orphan drugs: a scoping review and decision framework	Paulden MS, Tania; Menon, Devidas; McCabe, Christopher	2015
How to choose health technologies to be assessed by HTA? A review of criteria for priority setting	Specchia MLF, Marcella; Di Nardo, Francesco; Rotundo, Giovanna; Favaretti, Carlo; Ricciardi, Walter; de Waure, Chiara; Hta Working Group of the Italian Society of Hygiene, Preventive Medicine; Public, Health	2015
Public views on principles for health care priority setting: findings of a European cross-country study using Q methodology	van Exel JB, Rachel; Mason, Helen; Donaldson, Cam; Brouwer, Werner; EuroVa, Q. Team	2015
Ontario's approach to tackling drug funding sustainability	Anas RG, S.; McLeod, R.; McLaughlin, V.; Earle, C.; Arias, J.; Rey, M.; Jamal, H.	2016
Value-Based Assessment of New Medical Technologies: Towards a Robust Methodological Framework for the Application of Multiple Criteria Decision Analysis in the Context of Health Technology Assessment	Angelis AK, P.	2016
Application of a value framework based on multiple criteria decision analysis in the context of health technology assessment: A series of case studies with decision makers across European countries	Angelis AK, P.	2016
The Value of Medicines: A Crucial but Vague Concept	Antonanzas FT, Robert; Postma, Maarten	2016
Eight-year experience of using HTA in drug reimbursement: South Korea	Bae E-YH, Ji-Min; Kwon, Hye-Young; Jang, Suhyun; Lee, Hye-Jae; Bae, SeungJin; Yang, Bong-Min	2016

The influence of power and actor relations on priority setting and resource allocation practices at the hospital level in Kenya: a case study	Barasa EWC, Susan; English, Mike; Molyneux, Sassy	2016
Is there an alternative to quality-adjusted life years for supporting healthcare decision making?	Beresniak AD, Danielle	2016
Patients' perspectives can be integrated in health technology assessments: an exploratory analysis of CADTH Common Drug Review	Berglas SJ, Lauren; MacKean, Gail; Weeks, Laura	2016
Precision medicine and the changing role of regulatory agencies	Breckenridge AE, H. G.; Jarow, J. P.	2016
Trust, regulatory processes and NICE decision-making: Appraising cost-effectiveness models through appraising people and systems	Brown PH, Ferhana; Calnan, Michael	2016
Management of hospital formularies in Ontario: Challenges within a Local Health Integration Network	Burke NB, J. M.; Troyan, S.; Jegathisawaran, J.; Gosse, C.; Tonkin, M.; Kagoma, S.; Goeree, R.; Holbrook, A.	2016
The merry-go-round of approval, pricing and reimbursement of drugs against the Hepatitis C virus infection in Spain	Campillo-Artero CG-A, Sandra; Bernal-Delgado, Enrique	2016
Canadian Forum on Combined Organ Transplantation	Cantarovich MB-H, T. D.; Gill, J.; Tinckam, K.; Schiff, J.; Alwayn, I.; Bain, V.; Dipchand, A. I.; Isaac, D.; Kim, S. J.; Lien, D.; Zaltzman, J.; Young, K.; Nickerson, P.	2016
Competing Principles for Allocating Health Care Resources	Carter DG, Jason; Watt, Amber M.	2016
Governments Need Better Guidance to Maximise Value for Money: The Case of Australia's Pharmaceutical Benefits Advisory Committee	Carter DV, Arlene; Haji Ali Afzali, Hossein	2016
Use of programme budgeting and marginal analysis as a framework for resource reallocation in respiratory care in North Wales, UK	Charles JMB, G.; Thomas, K.; Johnstone, F.; Vandenblink, V.; Pethers, B.; Jones, A.; Edwards, R. T.	2016
Priority Setting Meets Multiple Streams: A Match to Be Further Examined? Comment on "Introducing New Priority Setting and Resource Allocation Processes in a Canadian Healthcare Organization: A Case Study Analysis Informed by Multiple Streams Theory	Cumming JM	2016
The International Right to Health: What Does It Mean in Legal Practice and How Can It Affect Priority Setting for Universal Health Coverage?	Dittrich RC, Leonardo; Gostin, Lawrence; Chalkidou, Kalipso; Li, Ryan	2016
Did It Matter That the Cancer Drugs Fund Was Not NICE? A Retrospective Review	Dixon PC, Charlotte; Hollingworth, William	2016
Are Indirect Benefits Relevant to Health Care Allocation Decisions?	Du Toit JM, Joseph	2016
Evaluation of the value of pharmaceutical products in Taiwan	Fang CHC, C. J.	2016
Multi-Criteria Decision Analysis for Assessment and Appraisal of Orphan Drugs	Iskrov GM-K, Tsonka; Stefanov, Rumen	2016
Economic Evaluations of Multicomponent Disease Management Programs with Markov Models: A Systematic Review	Kirsch F	2016

Potential impact of the implementation of multiple-criteria decision analysis (MCDA) on the Polish pricing and reimbursement process of orphan drugs	Kolasa KZ, Krzysztof M.; Kalo, Zoltan; Hermanowski, Tomasz	2016
Elements of orphan drugs value	Korchagina DM, A.; Toumi, M.; Falissard, B.	2016
How to Fairly Allocate Scarce Medical Resources: Ethical Argumentation under Scrutiny by Health Professionals and Lay People	Krutli PR, Thomas; Tornblom, Kjell Y.; Smieszek, Timo	2016
What makes a good economic evaluation in relapsed and refractory multiple myeloma: A review of reimbursement submissions to PCODR and nice	Li CMR, N. A.; Briggs, A.	2016
Stated and Revealed Preferences for Funding New High-Cost Cancer Drugs: A Critical Review of the Evidence from Patients, the Public and Payers	MacLeod TEH, Anthony H.; Mahal, Ajay	2016
Overview and comparison of frameworks for the valuation of oncology drugs	Maervoet JM, P.; Naidoo, S.	2016
Combining multi-criteria decision analysis and mini-health technology assessment: A funding decision-support tool for medical devices in a university hospital setting	Martelli NH, Paul; van den Brink, Helene; Boudard, Aurelie; Cordonnier, Anne-Laure; Devaux, Capucine; Pineau, Judith; Prognon, Patrice; Borget, Isabelle	2016
A quantitative analysis of health technology assessment (HTA) agencies for attributes driving transparency of the organization: Comparison of six Asia-pacific HTA agencies	Mazumder DK, A.; Gwatkin, N.; Medeiros, C.	2016
Understanding methodological differences between and the appropriate application of oncology value frameworks in healthcare decision-making	Monnickendam GM, K.; McKendrick, J.	2016
Use of the 'Accountability for Reasonableness' Approach to Improve Fairness in Accessing Dialysis in a Middle-Income Country	Moosa MRM, Jonathan David; Chirehwa, Maxwell T.; Benatar, Solomon R.	2016
Developing an evidence-based methodological framework to systematically compare HTA coverage decisions: A mixed methods study	Nicod EK, Panos	2016
SCIENTIFIC and SOCIAL VALUE JUDGMENTS for ORPHAN DRUGS in HEALTH TECHNOLOGY ASSESSMENT	Nicod EK, P.	2016
INTEGRATING HEALTH TECHNOLOGY ASSESSMENT PRINCIPLES IN FORMULARY MANAGEMENT	Teng MK, Ai Leng; Zhao, Ying Jiao; Lin, Liang; Lim, Boon Peng	2016
An Ethical Framework for Allocating Scarce Life-Saving Chemotherapy and Supportive Care Drugs for Childhood Cancer	Unguru YF, Conrad V.; Bernhardt, Brooke; Berg, Stacey; Pyke-Grimm, Kim; Woodman, Catherine; Joffe, Steven	2016

Administrative process and criteria ranking for drug entering health insurance list in Iran-TOPSIS-based consensus model	Viyanchi AG, A. R.; Rasekh, H. R.; Safikhani, H.	2016
Ethics in HTA: Examining the "Need for Expansion"	Abrishami PO, Wija; Hofmann, Bjorn	2017
Oncology drugs for orphan indications: how are HTA processes evolving for this specific drug category?	Adkins EMN, Lindsay; Floyd, David; Ratcliffe, Mark; Chevrou-Severac, Helene	2017
Stakeholder views on criteria and processes for priority setting in Norway: a qualitative study	Aidem JM	2017
Variation in Health Technology Assessment and Reimbursement Processes in Europe	Akehurst RLA, Eric; Renaudin, Noel; Sarkozy, Francois	2017
Prioritisation of specialist health care services; not NICE, not easy but it can be done	Anderson PW, Philip; Groves, Sam	2017
HIV prevention where it is needed most: Comparison of strategies for the geographical allocation of interventions: Comparison	Anderson SJG, P. D.; Ombam, R.; Hallett, T. B.	2017
Multiple Criteria Decision Analysis (MCDA) for evaluating new medicines in Health Technology Assessment and beyond: The Advance Value Framework	Angelis AK, P.	2017
Recommendations from the European Working Group for Value Assessment and Funding Processes in Rare Diseases (ORPH-VAL)	Annemans LA, Segolene; Le Cam, Yann; Facey, Karen; Gunther, Penilla; Nicod, Elena; Reni, Michele; Roux, Jean-Louis; Schlander, Michael; Taylor, David; Tomino, Carlo; Torrent-Farnell, Josep; Upadhyaya, Sheela; Hutchings, Adam; Le Dez, Lugdivine	2017
Economic evaluation of brentuximab vedotin for persistent hodgkin lymphoma	Babashov VB, M. A.; Mangel, J.; Zaric, G. S.	2017
Lives Saved Tool (LiST) costing: a module to examine costs and prioritize interventions	Bollinger LAS, Rachel; Winfrey, William; Adesina, Adebiyi	2017
Shouldn't nice cost-effectiveness thresholds be changing with the times?	Boodhna TH, J.	2017
Physicians' attitude towards selection of second line therapy with nilotinib and dasatinib in chronic myeloid leukemia patients	Breccia MB, M.; Rosti, G.; Cottone, F.; Cannella, L.; Guilhot, F.; Vignetti, M.; Efficace, F.	2017
Applying Multi-Criteria Decision Analysis (MCDA) Simple Scoring as an Evidence-based HTA Methodology for Evaluating Off-Patent Pharmaceuticals (OPPs) in Emerging Markets	Brixner DM, Nikos; Kalo, Zoltan; Hu, Shanlian; Shen, Jie; Wijaya, Kalman	2017

Payer Perspectives on Patient-Reported Outcomes in Health Care Decision Making: Oncology Examples	Brogan APD, Carla; Barrett, Amy M.; D'Alessio, Denise; Bal, Vasudha; Hogue, Susan L.	2017
Still Elegantly Muddling Through? NICE and Uncertainty in Decision Making About the Rationing of Expensive Medicines in England	Calnan MH, Ferhana; Brown, Patrick	2017
Societal perspective on access to publicly subsidised medicines: A cross sectional survey of 3080 adults in Australia	Chim LS, Glenn; Kelly, Patrick; Lipworth, Wendy; Hughes, Dyfrig A.; Stockler, Martin R.	2017
Three Sets of Case Studies Suggest Logic and Consistency Challenges with Value Frameworks	Cohen JTA, J. E.; Neumann, P. J.	2017
How Should Therapeutic Decisions about Expensive Drugs Be Made in Imperfect Environments?	Fleck LMD, Marion	2017
Don't Discount Societal Value in Cost-Effectiveness Comment on "Priority Setting for Universal Health Coverage: We Need Evidence-Informed Deliberative Processes, Not Just More Evidence on Cost-Effectiveness"	Hall W	2017
The cost-benefit of lung cancer care from a patient's perspective	Hicks DWB, G.; Addario, J.	2017
How have systematic priority setting approaches influenced policy making? A synthesis of the current literature	Kapiriri LR, Donya	2017
National reimbursement listing determinants of new cancer drugs: a retrospective analysis of 58 cancer treatment appraisals in 2007-2016 in South Korea	Kim E-SK, Jung-Ae; Lee, Eui-Kyung	2017
New treatments for serious conditions: ethical implications	King NMPB, C. E.	2017
Ethical Challenges in the Provision of Dialysis in Resource-Constrained Environments	Luyckx VAM, Ingrid; Ejigu, Addisu M.; Moosa, M. Rafique	2017
Financial Toxicity of Cancer Drugs: Possible Remedies from an Ethical Perspective	Marckmann GIdS, Jurgen	2017
Applying the institute for clinical and economic review's (ICER) methodology to Canada: An improved way to take into consideration drug affordability challenges?	Mouallif SC-R, S.; Barbeau, M.	2017
Dealing with Uncertainty and Accounting for Social Value Judgments in Assessments of Orphan Drugs: Evidence from Four European Countries	Nicod EBB, Karen; Durand-Zaleski, Isabelle; Kanavos, Panos	2017
The role of patient groups in nice technology appraisals for drugs for rare diseases	Pandolfo RS, S.	2017
Preferences of the general public for reimbursement criteria for expensive drugs: A multi criteria decision analysis for rituximab and bevacizumab	Van Steen CC, E.; Treur, M.; Verbeek, N. A.; Franken, M.	2017
P&T Committee drug prioritization criteria: A tool developed by a saudi health care system	Abu Esba LDA, H.; Alhammad, A.; Ferwana, M.; Yousef, C.; Ismail, S.	2018
A Review of the Impacts of Different Approaches for Diabetes Prevention and a Framework for Making Investment Decisions	Alva ML	2018

Using health technology assessment to assess the value of new medicines: results of a systematic review and expert consultation across eight European countries	Angelis AL, Ansgar; Kanavos, Panos	2018
Liver transplantation for critically ill cirrhotic patients: Overview and pragmatic proposals	Artzner TM, Baptiste; Besch, Camille; Levesque, Eric; Faitot, Francois	2018
Role of economic evidence in coverage decision-making in South Korea	Bae E-YK, Hui Jeong; Lee, Hye-Jae; Jang, Junho; Lee, Seung Min; Jung, Yunkyung; Yoon, Nari; Kim, Tae Kyung; Kim, Kookhee; Yang, Bong-Min	2018
Multi-Criteria Decision Analysis (MCDA) Models in Health Technology Assessment of Orphan Drugs-a Systematic Literature Review. Next Steps in Methodology Development?	Baran-Kooiker AC, Marcin; Kooiker, Coen	2018
Qualitative analysis of national documents on health care services and pharmaceuticals` purchasing challenges: evidence from Iran	Bastani PS, Mahnaz; Dinarvand, Rassoul; Kashefian-Naeeini, Sara; Vatankhah, Soudabeh	2018
Trade-offs, fairness, and funding for cancer drugs: key findings from a deliberative public engagement event in British Columbia, Canada	Bentley CC, Sarah; Burgess, Michael M.; Regier, Dean; McTaggart-Cowan, Helen; Peacock, Stuart J.	2018
A multicriteria resource allocation model for the redesign of services following birth	Bowers JC, Helen; Mould, Gillian; Miller, Martin; Page, Miranda; Harris, Fiona; Bick, Debra	2018
An Evidence Framework for Off-Patent Pharmaceutical Review for Health Technology Assessment in Emerging Markets	Brixner DK, Zoltan; Maniadakis, Nikos; Kim, Kyoo; Wijaya, Kalman	2018
The meaning of severity - do citizens views correspond to a severity framework based on ethical principles for priority setting?	Broqvist MS, Lars; Garpenby, Peter; Krevers, Barbro	2018
Little to lose and no other options: Ethical issues in efforts to facilitate expanded access to investigational drugs	Bunnik EMA, N.; van de Vathorst, S.	2018
Disinvestment in healthcare: an overview of HTA agencies and organizations activities at European level	Calabro GELT, G.; de Waure, C.; Villari, P.; Federici, A.; Ricciardi, W.; Specchia, M. L.	2018
Prices For Common Cardiovascular Drugs In The US Are Not Consistently Aligned With Value	Campbell JDB, Vasily; Whittington, Melanie D.; Rubin, Robert J.; Raggi, Paolo; Briggs, Andrew H.	2018
Rare diseases under different levels of economic analysis: current activities, challenges and perspectives	Cannizzo SL, Valentina; Palla, Ilaria; Pirri, Salvatore; Trieste, Leopoldo; Triulzi, Isotta; Turchetti, Giuseppe	2018
Fair, just and compassionate: A pilot for making allocation decisions for patients requesting experimental drugs outside of clinical trials	Caplan ALT, J. Russell; Kearns, Lisa; Bateman-House, Alison S.; Mitchell, Edith; Arawi, Thalia; Upshur, Ross; Singh, Ilina; Rozynska, Joanna; Cwik, Valerie; Gardner, Sharon L.	2018

Societal values and child health priorities: Empirical evidence for paediatric cancer drug policy	Denburg AC, S.; Abelson, J.; Hurley, J.; Ungar, W.	2018
Social values and public policy on cancer drug funding decisions for children in Canada	Denburg AG, M.; Ungar, W.; Abelson, J.	2018
Decision-making criteria for medicine reimbursement in Slovenia: an expert panel discussion	Deticek AJ, Andrej; Locatelli, Igor; Kos, Mitja	2018
There is always a better way: Managing uncertainty in decision making about new cancer drugs in Canada	Driedger SMC, Elizabeth; Annable, Gary; Brouwers, Melissa	2018
COMPARISON OF PHARMACOECONOMIC (PE) EVALUATIONS FOR DRUGS FOR RARE DISEASES (DRDS) EVALUATED BY CADTH AND NICE	Gosain SB, A.; Kirby, J.; Ralph, L.; Lilley, C.; Lawrence, D.	2018
Action Guide for Addressing Ethical Challenges of Resource Allocation Within Community-Based Healthcare Organizations	Harrison KLT, Holly A.; Merritt, Maria W.	2018
Orphan Drugs: Health Technology Assessment and Pricing in Europe	Jaroslawski ST, M.; Korchagina, D.; Jadot, G.	2018
THE FEASIBILITY OF APPLYING THE MULTIPLE CRITERIA DECISION ANALYSIS FOR THE EVALUATION OF MEDICAL DEVICES IN THE CONTEXT OF HOSPITAL-BASED HEALTH TECHNOLOGY ASSESSMENT IN TURKEY	Kocaman MT, C. G.; Ozer, E.; Cakmak, H. S.; Baysu, F.; Kahveci, R.	2018
Revealed preferences towards the appraisal of orphan drugs in Poland - multi criteria decision analysis	Kolasa KZ, Krzysztof Miroslaw; Zah, Vladimir; Kalo, Zoltan; Lewandowski, Tadeusz	2018
The development of funding recommendations for health technologies at the state level: A South Australian case study	Lambert RC, Drew; Burgess, Naomi; Haji Ali Afzali, Hossein	2018
Value assessment in oncology drugs: funding of drugs for metastatic breast cancer in Canada	Lemieux JA, S.	2018
RELATIVE VALUE OF EVIDEM MCDA FRAMEWORK FOR REFLECTIVE DRUG EVALUATION AMONG HOSPITAL PHARMACISTS IN PORTUGAL	Mirco AP, E.; Farinha, M. H.; Gil, A.; Shepherd, J.; Badia, X.	2018
Decision-making frameworks and considerations for informing coverage decisions for healthcare interventions: a critical interpretive synthesis	Morgan RLK, L.; Guyatt, G. H.; Johnson, A.; Lavis, J. N.	2018
Will the reformed Cancer Drugs Fund address the most common types of uncertainty? An analysis of NICE cancer drug appraisals	Morrell LW, Sarah; Schuh, Anna; Middleton, Mark R.; Rees, Sian; Barker, Richard W.	2018
Evaluating and Valuing Drugs for Rare Conditions: No Easy Answers	Ollendorf DAC, Richard H.; Pearson, Steven D.	2018

MULTI-CRITERIA DECISION ANALYSIS AS A DECISION-SUPPORT TOOL FOR DRUG EVALUATION: A PILOT STUDY IN A PHARMACY AND THERAPEUTICS COMMITTEE SETTING	Roldan UBB, Xavier; Marcos-Rodriguez, Jose Antonio; de la Cruz-Merino, Luis; Gomez-Gonzalez, Jaime; Melcon-de Dios, Ana; Caraballo-Camacho, Maria de la O.; Cordero-Ramos, Jaime; Alvarado-Fernandez, Maria Dolores; Galiana-Auchel, Jose Manuel; Calleja-Hernandez, Miguel Angel	2018
The Relative Importance of Clinical, Economic, Patient Values and Feasibility Criteria in Cancer Drug Reimbursement in Canada: A Revealed Preferences Analysis of Recommendations of the Pan-Canadian Oncology Drug Review 2011-2017	Skedgel CW, Dominika; Hu, Min	2018
Accountable and reasonable processes for coverage decision-making for rare disease and regenerative therapies-which criteria should be considered and why?	Wagner MS, D.; Casciano, R.; Brougham, M.; Petrie, C. D.; Abrishami, P.; Avouac, B.; Kind, P.; Sarria-Santamera, A.; Schlander, M.; Mantovani, L. G.; Tringali, M.	2018
Towards a Transparent, Credible, Evidence-Based Decision-Making Process of New Drug Listing on the Hong Kong Hospital Authority Drug Formulary: Challenges and Suggestions	Wong CKHW, Olivia; Cheung, Bernard M. Y.	2018
Stakeholder driven development of a multi-criteria decision analysis tool for purchasing off-patent pharmaceuticals in Kuwait	Abdullah AHH, A. P.; Al-Hussaini, M.; Lemay, J.; Alowayesh, M.; Kalo, Z.	2019
In the Era of Cost-Effectiveness Analysis, Affordability Is a Limiting Factor for Patients' Access to Innovative Cancer Treatments	Aguiar PN, Jr.; Adashek, Jacob J.; Roitberg, Felipe; Noia Barreto, Carmelia Maria; Del Giglio, Auro; Lopes, Gilberto L., Jr.	2019
PMU114 SPECIALISED HEALTH TECHNOLOGY ASSESSMENT PROCESSES FOR VERY RARE DISEASES: PAST, PRESENT AND FUTURE	Akehurst RLM, J. A.; Jones, E.; Mason, N.; Schurer, M.; Chapman, A. M.; Horscroft, J.	2019
PRO116 UNDERSTANDING THE ROLE OF REAL-WORLD EVIDENCE IN HEALTH TECHNOLOGY ASSESSMENT FOR ORPHAN DRUGS	Akesson CL, S.; Bagshaw, E.; Kousoulakou, H.; Larkin, M.	2019
A Framework for Aiding the Translation of Scientific Evidence into Policy: The Experience of a Hospital-Based Technology Assessment Unit	Almeida NDM, Lorraine; Nicolau, Ioana; Sinclair, Alison; Forero, David Felipe; Brophy, James M.; Mayo, Nancy; Dendukuri, Nandini	2019
Patient involvement in reflective multicriteria decision analysis to assist decision making in oncology	Badia XA, Alfonso; Fernandez, Ana; Gimon, Antonia; Nafria, Begona; Gaspar, Bernard; Guarga, Laura; Galvez,	2019

	Maria; Fuentes, Marta; Paco, Noelia; Saldana, Roberto	
Role of Health Technology Assessment in Drug Policies: Korea	Bae E-Y	2019
Applicability of the EVIDEM multi-criteria decision analysis framework for orphan drugs N results from a study in 7 Eurasian countries	Baran-Kooiker AA, K.; Gaitova, K.; Holownia-Voloskova, M.; Turcu-Stiolica, A.; Kooiker, C.; Piniazhko, O.; Czech, M.	2019
Patient empowerment and access to medicines: Insights from a scientist-patient suffering from erythropoietic protoporphyria	Barman-Aksozen J	2019
Addressing the affordability of cancer drugs: using deliberative public engagement to inform health policy	Bentley CP, Stuart; Abelson, Julia; Burgess, Michael M.; Demers-Payette, Olivier; Longstaff, Holly; Tripp, Laura; Lavis, John N.; Wilson, Michael G.	2019
Qualitative Exploration of Triangulated, Shared Decision-Making in Rheumatoid Arthritis	Binder-Finnema PD, K.; Hsiao, B.; Fraenkel, L.	2019
Getting to the Table: Changing Ideas about Public and Patient Involvement in Canadian Drug Assessment	Boothe K	2019
Two's company, is three a crowd? Ethical cognition in decision making and the role of industry third parties in pediatric diabetes care	Cameron FJM, Bryanna; Gillam, Lynn	2019
What's fair to an individual is not always fair to a population: A qualitative study of patients and their health professionals using the Cancer Drugs Fund	Chamberlain CO-S, Amanda; MacKichan, Fiona; Donovan, Jenny L.; Hollingworth, William	2019
Community views on factors affecting medicines resource allocation: cross-sectional survey of 3080 adults in Australia	Chim LS, Glenn; Kelly, Patrick J.; Lipworth, Wendy; Hughes, Dyfrig A.; Stockler, Martin R.	2019
Doctors' perceptions of how resource limitations relate to futility in end-of-life decision making: a qualitative analysis	Close EW, Ben P.; Willmott, Lindy; Gallois, Cindy; Parker, Malcolm; Graves, Nicholas; Winch, Sarah	2019
Public perspectives on disinvestments in drug funding: results from a Canadian deliberative public engagement event on cancer drugs	Costa SB, Colene; Regier, Dean A.; McTaggart-Cowan, Helen; Mitton, Craig; Burgess, Michael M.; Peacock, Stuart J.	2019
Too Many Patients...A Framework to Guide Statewide Allocation of Scarce Mechanical Ventilation During Disasters	Daugherty Biddison ELF, Ruth; Gwon, Howard S.; Mareiniss, Darren P.; Regenberg, Alan C.; Schoch-Spana, Monica; Schwartz, Jack; Toner, Eric S.	2019

A weighted criterion-based approach to value assessment of oncology drugs	Ezeife DAC, E. R.; Fares, A. F.; Sung, M.; Dionne, F.; Mitton, C.; Earle, C.; Chan, K. K.; Leighl, N. B.	2019
Drug Treatment Value in a Changing Oncology Landscape: A Literature and Provider Perspective	Frois CH, Andrew; Jarvis, John; Grice, Kathryn; Wong, Ken; Zacker, Christopher; Sasane, Rahul	2019
Implementing reflective multicriteria decision analysis (MCDA) to assess orphan drugs value in the Catalan Health Service (CatSalut)	Guarga LB, Xavier; Obach, Merce; Fontanet, Manel; Prat, Alba; Vallano, Atonio; Torrent, Josep; Pontes, Caridad	2019
International experiences in multicriteria decision analysis (MCDA) for evaluating orphan drugs: a scoping review	Lasalvia PP-P, L.; Moreno, M.; Castrillon, J.; Romano, G.; Garzon-Orjuela, N.; Rosselli, D.	2019
Integrating public preferences into reimbursement decisions: Case studies from Belgium and New Zealand	Leopold CL, C. Y.; Wagner, A. K.	2019
Incorporating Affordability Concerns Within Cost-Effectiveness Analysis for Health Technology Assessment	Lomas JRS	2019
Using a stated preference discrete choice experiment to assess societal value from the perspective of patients with rare diseases in Italy	Lopez-Bastida JR-G, Juan Manuel; Aranda-Reneo, Isaac; Taruscio, Domenica; Magrelli, Armando; Kanavos, Panos	2019
Using a stated preference discrete choice experiment to assess societal value from the perspective of decision-makers in Europe. Does it work for rare diseases?	Lopez-Bastida JR-G, J. M.; Aranda-Reneo, I.; Trapero-Bertran, M.; Kanavos, P.; Rodriguez Martin, B.	2019
What Matters Most for Treatment Decisions in Hepatitis C: Effectiveness, Costs, and Altruism	Mattingly TJS, J. F.; Perfetto, E. M.; Kottilil, S.; Mullins, C. D.	2019
What is driving HTA decision-making? Evidence from cancer drug reimbursement decisions from 6 European countries	Maynou LC, John	2019
Investigating the relative value of health and social care related quality of life using a discrete choice experiment	Mulhern BN, Richard; De Abreu Lourenco, Richard; Malley, Juliette; Street, Deborah; Viney, Rosalie	2019
Ethical considerations relating to healthcare resource allocation decisions	Olver ID, Susan; Kenner, Jeremy; Kerridge, Ian; McGovern, Kevin; Milligan, Eleanor; Mortimer, Robin; Australian Health Ethics Committee of the National, Health; Medical Research, Council	2019
Defining the Value of Health Technologies in Latin America: Developments in Value Frameworks to Inform the Allocation of Healthcare Resources	Pichon-Riviere AG-M, Sebastian; Oortwijn, Wija; Augustovski, Federico; Sampietro-Colom, Laura	2019

High cost drug policies in Canadian children's hospitals	Rieder MP, A.	2019
Evaluating Canadians' Values for Drug Coverage Decision Making	Rizzardo SB, Nick; Dragojlovic, Nick; Douglas, Conor; Li, Kathy H.; Mitton, Craig; Marra, Carlo; Blanis, Litsa; Lynd, Larry D.	2019
Analysis of Health Technology Assessments of Orphan Drugs in Ireland from 2012 to 2017	Usher CM, Laura; Tilson, Lesley; Barry, Michael	2019
Impact of health technology assessment and managed entry schemes on reimbursement decisions of centrally authorised medicinal products in Belgium	Van Wilder PP, Magali; Dupont, Alain	2019
Multiple Criteria Decision Analysis for HTA across four EU Member States: Piloting the Advance Value Framework	Angelis AL, M.; Montibeller, G.; Molina-Lopez, T.; Zawada, A.; Orzel, K.; Arickx, F.; Espin, J.; Kanavos, P.	2020
Severity as a Priority Setting Criterion: Setting a Challenging Research Agenda	Barra MB, Mari; Gustavsson, Erik; Henriksson, Martin; Juth, Niklas; Sandman, Lars; Solberg, Carl Tollef	2020
Non-NHS funded therapy versus clinical trials: the importance of patient autonomy in mesothelioma treatment decision making	Bolton SS, J.; Swaboda, J.	2020
Developing a framework to incorporate real-world evidence in cancer drug funding decisions: the Canadian Real-world Evidence for Value of Cancer Drugs (CanREValue) collaboration	Chan KN, Seungree; Evans, Bill; de Oliveira, Claire; Chambers, Alexandra; Gavura, Scott; Hoch, Jeffrey; Mercer, Rebecca E.; Dai, Wei Fang; Beca, Jaclyn; Tadrous, Mina; Isaranuwatchai, Wanrudee	2020
Access to treatment and the constitutional right to health in Germany: a triumph of hope over evidence?	Ettelt S	2020
Beyond cost-effectiveness: A five-step framework for appraising the value of health technologies in Asia-Pacific	Finkelstein EAK, Anirudh; Doble, Brett	2020
Using GRADE methodology to assess innovation of new medicinal products in Italy	Fortinguerra FT, G.; Trotta, F.; Addis, A.	2020
Developing the ethical framework of end-stage kidney disease care: from practice to policy	Luyckx VAM, D. E.; Moosa, M. R.; Bello, A. K.; Bellorin-Font, E.; Chan, T. M.; Claure-Del Granado, R.; Douthat, W.; Eiam-Ong, S.; Eke, F. U.; Goh, B. L.; Jha, V.; Kendal, E.; Liew, A.; Mengistu, Y. T.; Muller, E.; Okpechi, I. G.; Rondeau, E.; Sahay, M.; Trask, M.; Vachharajani, T.	2020

Social values for health technology assessment in Canada: a scoping review of hepatitis C screening, diagnosis and treatment	O'Keefe-Markman CL, Kristina Dawn; McCabe, Christopher; Hyshka, Elaine; Bubela, Tania	2020
Economic Evaluation for Pricing and Reimbursement of New Drugs in Spain: Fable or Desideratum?	Oliva-Moreno JP-J, Jaume; Trapero-Bertran, Marta; Epstein, David; Pinyol, Carme; Sacristan, Jose Antonio	2020
Development of the WHO-INTEGRATE evidence-to-decision framework: an overview of systematic reviews of decision criteria for health decision-making	Stratil JMB, R.; Scheel, I.; Nacken, A.; Rehfuess, E. A.	2020
Determinants of Orphan Drug Prices in Germany	Worm FD, C. M.	2020
