# Supplementary material for: Institutional Priority-Setting for Novel Drugs and Therapeutics: A Qualitative Systematic Review
Source: Int J Health Policy Manag. 2024 Feb 10;13:7494. doi: 10.34172/ijhpm.2024.7494 (PMC11016276; doi:10.34172/ijhpm.2024.7494)
Supplement: Supplementary file 5 — Summary of Identified Themes, Conclusions and Challenges From Literature Analysis. [file ijhpm-13-7494-s005.pdf]

Article title: Institutional Priority-Setting for Novel Drugs and Therapeutics: A Qualitative Systematic Review

Journal name: International Journal of Health Policy and Management (IJHPM)

Authors' information: Daniel E. Wang[1], Maram Hassanein[2], Yasmeen Razvi[3,4], Randi Zlotnik Shaul[1,2,4], Avram Denburg[1,4,5]*

[1]Department of Paediatrics, University of Toronto, Toronto, ON, Canada.

[2]Department of Bioethics, The Hospital for Sick Children, Toronto, ON, Canada.

[3]Temerty Faculty of Medicine, University of Toronto, Toronto, ON, Canada.

[4]Child Health Evaluative Sciences, SickKids Research Institute, Toronto, ON, Canada.

[5]Division of Paediatric Haematology/Oncology, The Hospital for Sick Children, Toronto, ON, Canada.

***Correspondence to:** Avram Denburg; Email: avram.denburg@sickkids.ca

Citation: Wang DE, Hassanein M, Razvi Y, RZ Shaul, Denburg A. Institutional priority-setting for novel drugs and therapeutics: a qualitative systematic review. Int J Health Policy Manag. 2024;13:7494. doi:10.34172/ijhpm.2024.7494

Supplementary file 5. Summary of Identified Themes, Conclusions and Challenges From Literature Analysis

Category	Subject	Theme	Topic	Conclusion or Challenge	Description
A. Institutional Strategy	A.1. Corporate Vision	A.1.A. The Importance of Strategic Alignment: Any funding decision should be made with consideration for the institution's broader strategic goals, not only with respect to the specific disease being targeted, but its overall economic goals, local and regional state of health, and institutional culture especially with respect to equity and inclusion and other social values.	A.1.1. Strategic Goals	A.1.1.1. Conclusion: Strategic Relevance	Institutional policies must align to strategic goals, values, and objectives.
		A.1.B. The Importance of Fueling Innovation: Many institutions are explicitly trying to promote scientific innovation through healthcare funding decisions although such innovation and its benefits can be difficult to quantify or evaluate at the time of decision-making.	A.1.2. Innovation	A.1.2.1. Conclusion: Importance of Innovation	Innovation increasing becoming an important consideration - both to support novel therapeutics but also to help mobilize interest and broader public / government support for research into areas that may otherwise be underserved.
B. Substantive Criteria	B.1. Anticipated Outcomes	B.1.A. The Importance of Robust Evidence: Best available evidence, preferably from scientifically robust sources, is critical to well-	B.1.1. Evidence	B.1.1.1. Conclusion: Best	Almost all decision-makers place a significant emphasis on using the best available evidence to inform their decisions.

Category	Subject	Theme	Topic	Conclusion or Challenge	Description
		informed funding decision but may require non-literature sources for health areas that are not yet well supported by academic literature, e.g. patient reported outcomes (PROs) and real-world evidence (RWE).		Available Evidence	
				B.1.1.2. Conclusion: Importance of Real-World Evidence	Real-World Evidence (RWE), like patient reported outcomes (PROs), is an important and valuable source of information to both validate clinical trial / research data as well as to inform ongoing assessments and evaluation of prior decisions.
				B.1.1.3. Conclusion: Limited Evidence A Common Source of Uncertainty	Limited availability of scientific evidence, especially for rare diseases or novel therapeutics, is a common source of uncertainty, which places more emphasis on proper appraisal of evidence as well as trying to look for other sources of corroborative data.
		B.1.B. The Usefulness of Objective Data: Using quantifiable information, when possible, helps stakeholders compare health or economic data and using peer-reviewed data quality assessment methods such as GRADE can facilitate efficient and effective data reviews as part of overall funding decisions.		B.1.1.4. Conclusion: Patient Reported Outcomes Are Valuable	Patient Reported Outcomes (PROs) can help with real-world evaluation and provide non-trial related but valuable data for decision-makers.
				B.1.1.5. Conclusion: Quality Supporting Evidence	Many decision-makers look at quality assessment systems such as GRADE to help decision-makers understand the quality of supporting evidence.
				B.1.1.6. Conclusion: Sources of Evidence Vary	Sources of evidence to support decision-making varies, preferably from clinical trials but if they lack such trials, then other sources include lesser quality sources such as systematic review, literature review, observational studies, and in some cases manufacturing or industry data as well as patient reported outcomes (PROs).
		B.1.C. The Importance of Defining Health Benefits: While definitions may vary by institution as to the health "benefit" of an intervention, it is clear that however it is defined, anticipated health benefit of an	B.1.2. Expertise	B.1.2.1. Conclusion: Clinical Expertise From Clinicians or Government Agencies	Medical / clinical expertise is usually represented by clinicians and / or government bodies with clinical or regulatory oversight.

Category	Subject	Theme	Topic	Conclusion or Challenge	Description
		intervention is a critical factor in funding decisions.	B.1.3. Health Benefits	B.1.3.1. Conclusion: Importance of Magnitude of Benefit	Not only individual benefit is important, but the magnitude of overall benefit is important - often measured or estimated by patient population size.
				B.1.3.2. Conclusion: Comparative Effectiveness	Most assessments of drugs or therapeutics include a comparative assessment of the current standard or alternative treatment options to highlight effectiveness of the proposed intervention.
		B.1.D. The Importance of Objective Health Benefits: Many institutions define health benefit as some combination of patient quality of life, size of patient population, and treatment accessibility but the more objective or quantifiable, the easier it is to compare, especially when considering the economic costs and benefits of an intervention.		B.1.3.3. Conclusion: Individual QOL Benefit	Most studies identify quality of life (QOL) as a key health benefit to be expected as a result of the treatment or therapeutic intervention for a given patient.
				B.1.3.4. Conclusion: Quantifying Health Benefit	Many decision-makers use quantified tools / methods to describe the benefit of interventions e.g. quality adjusted life years (QALY), disability adjusted life years (DALY) to enable cross-illness comparisons.
		B.1.E. The Importance of Defining Alternatives: It is commonly accepted that benefits of an intervention must be compared to both existing as well as planned alternatives, in order to fully understand the health as well as economic impact of a planned intervention.		B.1.3.5. Conclusion: Treatment Ease	Studies and decision-makers are increasingly looking at identifying easing the treatment administration e.g. less frequent dosing, increased compliance, or decreased payer cost.
			B.1.4. Health Risks	B.1.4.1. Conclusion: Importance of Quality Assurance	Quality assurance of the drug or treatment manufacturing process is an important consideration, seemingly more so in underdeveloped geographies.
				B.1.4.2. Conclusion: Safety = Tolerability + Contraindication s	Safety profile can include both patient tolerability as well as the extent of contraindications for drug / treatment administration.
		B.1.F. The Importance of Identifying Health Risks: Whether called "safety", "tolerability", "contraindications" or other risk-related		B.1.4.3. Conclusion:	Severity of disease is a key consideration - often measured by mortality, quality of life (QOL), quality

Category	Subject	Theme	Topic	Conclusion or Challenge	Description
		impacts of an intervention, the health-related drawbacks of an intervention is "must have" information in the decision-making process for a well-informed decision.		Severity of Disease	adjusted life years (QALY) or other indicator, usually in comparison to the alternative intervention.
				B.1.4.4. Conclusion: Side Effect Profile of Proposed Treatment	Safety and side effects of proposed treatment is a de facto consideration in decision-making - the challenge lies in quantifying and / or collecting source data.
	B.2. Need	B.2.A. The Importance of Intervention Need: Like health benefits and health risks, the definition for intervention need is not always the same (usually a combination of disease severity, disease prevalence, burden of disease and availability of alternative treatments) but the concept of defining the requirement for a new intervention is a critical part of healthcare funding decisions and forms the initial basis upon which the data collection and analysis occurs.	B.2.1. Availability of Alternatives	B.2.1.1. Challenge: Accessibility Not Equal to Availability	Although availability of alternatives is an important criterion in decision-making, accessibility - often defined as the patient's ability to obtain the alternative intervention, and affordability - defined as the patient's ability to pay for the alternative relative to their income - are not always included in the concept of availability.
				B.2.1.2. Challenge: Difficulty of Measuring Alternatives	Some authors have commented on the difficulty of comparing alternative treatments, especially when real-world evidence (RWE) or patient reported outcomes (PROs) are lacking. Bioequivalence studies are helping to address this challenge.
				B.2.1.3. Conclusion: Unmet Need / Importance of Alternate Treatments	The availability of alternative treatments is an important decision criterion when determining the medical necessity or medical importance of an intervention.
			B.2.2. Disease Burden	B.2.2.1. Challenge: Rarity vs Disease Prevalence	There are contrasting views on whether rarity or high prevalence should be more important in system-level decision-making as the former promotes equity and accessibility while the latter promotes value for money and perhaps is socially or politically more acceptable.
				B.2.2.2. Conclusion: Prevalence or Population Size	The target size of disease population or prevalence is a key criterion for decision-makers - especially as it related to value for money principles.

Category	Subject	Theme	Topic	Conclusion or Challenge	Description
		B.2.B. The Conflict Between Rare and Common: Institutions recognize that the polarity between diseases that are common and those that are rare include different data and assumptions about their associated cost of treatments – often driven by research dollars, political appetite, and patient group advocacy – and are increasingly being explicitly acknowledged in funding decisions by using different economic valuations, explicit inclusion of social justice criteria in decision-making, or having separate funds for different groups of illnesses.		B.2.2.3. Conclusion: Rarity as Important Factor	Many authors also consider rarity as an important equity criterion - somewhat in contrast to disease prevalence as a historically important criterion.
				B.2.2.4. Conclusion: Burden of Disease	Burden of disease, which is not always well defined, is an important decision criterion and most often relates to the concept of "severity" - considering quality of life, mortality, morbidity, and life expectancy.
				B.2.2.5. Conclusion: Category of Disease or Vulnerable Groups	Some decision-makers include the category of disease as a decision criteria e.g. "vulnerable populations" such as elderly and children. Whether a disease is paediatric relevant is considered important by some authors to "improve access and adoption" {art 125}, or by the population at large {art 73] in Australia.
				B.2.2.6. Conclusion: Vague and Multiple Definitions of Disease Burden	Burden has no universal definition and often blurs concepts relating to "need", "severity", "prevalence" - but almost always seems to be used in a biomedical context (as opposed to financial or economic concept).
		B.2.C. The Importance of Real-World Evidence to Support Treatment Need: In addition to the importance of providing Real-World Evidence (RWE) or Patient Reported Outcomes (PROs) to help with ongoing monitoring and evaluation of previous funding decisions, RWE and PROs can help fill a void in scientific literature on the need for a novel intervention, especially for rare or orphan diseases or other diseases with special or limited population sizes.		B.2.2.7. Conclusion: Burden = Severity + Prevalence	Burden of illness can be described as the combination of severity of illness and prevalence of disease in the population.
				B.2.2.8. Conclusion: Severity as Important Criteria	Severity - a combination of healthy quality of life (hQOL), assessment of life-threatening condition and survival or disease progression - is used by almost all decision-makers for the purchase or support of therapeutic interventions.
				B.2.2.9. Conclusion: Severity Given	In some decision-making institutions (e.g. NICE), disease severity is afforded a higher threshold (e.g. ICER) in decision-making as a way to provide equity across different diseases.

Category	Subject	Theme	Topic	Conclusion or Challenge	Description
				Higher Importance	
				B.2.2.10. Conclusion: Indication Uniqueness	Some decision-makers place importance on the uniqueness of the indication for intervention (e.g. rareness) as a measure to promote healthcare system equity.
	B.3. Financial Considerations	B.3.A. The Prevalence of Direct and Indirect Costs: Direct and indirect costs are commonly used in healthcare funding decisions as financial impacts on care-givers, broader healthcare system, in addition to the patients themselves, are important considerations especially at regional or national healthcare levels.	B.3.1. Direct Financial Considerations	B.3.1.1. Challenge: Do Paediatric Patients Incur Greater Costs	Some decision-makers consider the patient cost per year but if paediatric patients have longer illness time horizons, how do their lifetime costs get considered?
				B.3.1.2. Challenge: What is More Important Cost-Effectiveness or Disease Severity	Some decision-makers place higher priority on cost-effectiveness (utilitarianism) than disease severity but social considerations may also be taken into account elsewhere (social initiatives, equity programs etc.).
				B.3.1.3. Conclusion: Direct Cost to Patients Is Paramount	The direct cost of the intervention is almost always a key consideration whereas purchase cost (i.e. a broader cost to society) is not always considered.
		B.3.B. The Prevalence and Diversity of Economic Modeling: While Cost Effectiveness Analysis (CEA), Incremental Cost Effectiveness Ratio (ICER) and Budget Impact Analysis (BIA) are commonly used economic tools to help compare the financial impacts of different treatment options, they all have benefits and drawbacks and none do a particularly effective job at capturing broader socioeconomic impacts.		B.3.1.4. Conclusion: Direct Costs Economic Models and Tools Commonly Used	Many decision-makers are using budgeting or financial tools to assist with decision-making including budgeting or costing tools such as Budget Impact Analysis (BIA), Cost Effectiveness Analysis (CEA) or Cost-Benefit Analysis (CBA).
				B.3.1.5. Conclusion: Importance of Appraising Economic Data	Like clinical or scientific data, economic data should also be evaluated objectively and rigorously - although it seems that this is not often done as well as for scientific data.

Category	Subject	Theme	Topic	Conclusion or Challenge	Description
			B.3.2. Indirect Financial Considerations	B.3.2.1. Conclusion: Broader Socioeconomic Considerations	Other socioeconomic benefits can include job creation, avoiding extra costs, promoting innovation, etc.
				B.3.2.2. Conclusion: Importance of Caregiver Costs	The financial costs to caregivers is also important in terms of time away from work, as well as travel or other costs.
		B.3.C. The Importance of Affordability: Decision-makers are increasingly looking at not just cost but affordability of a healthcare intervention, which speaks to the importance of social justice and accessibility of disadvantaged populations as well as the rising cost of rare illness treatments.		B.3.2.3. Conclusion: Healthcare System Costs Are Also Important	Indirect or broader system costs such as the impact to broader healthcare system costs are sometimes considered by decision-makers, more likely those in positions of policy or regulatory authority.
				B.2.3.4. Conclusion: Importance of Direct but Non-Medical Costs	Other direct non-medical costs used in decision-making include fees, drug supply costs, manufacturing costs, education and outreach costs to promote new treatments, etc.
			B.3.3. Economic Tools	B.3.3.1. Conclusion: BIA Incorporate Affordability but CEA Does Not	Affordability is an increasing growing consideration in economic analyses that are not typically captured in cost effectiveness analyses but are in budget impact analyses (BIA).
				B.3.3.2. Conclusion: The Challenges of Cost Effectiveness Ratios	Cost effective analysis (CEA) and Incremental Cost Effectiveness Ratio (ICER) do not always consider affordability, nor indirect costs or broader socioeconomic costs.
				B.3.3.3. Conclusion: Common Economic Tools	Commonly used economic tools include Business Impact Analysis (BIA), Cost Benefit Analysis (CBA), Cost effective analysis (CEA) and Incremental Cost Effectiveness Ratio (ICER), although these are continuously being enhanced or modified

Category	Subject	Theme	Topic	Conclusion or Challenge	Description
					depending on the usage and utility to various decision-makers.
	B.4. Societal Interests	B.4.A. The Importance of Social Values: Most government or publicly-funded institutions consider social justice and societal concerns integral criteria in healthcare decision-making but it can be challenging to address different values that stakeholders place on different social drivers and especially difficult to quantify in economic terms on a timely basis; this decision criteria may become even more important depending on the social / political climate.	B.4.1. Social Justice	B.4.1.1. Conclusion: Importance of Equity of Access	Equity is often measured by the ability for underserviced populations to access available treatments and interventions. Some decision-makers place a higher priority on enabling access to such populations.
				B.4.1.2. Conclusion: Rarity May Address Social Inequities	Focusing on rare diseases may help address social inequities by turning the focus to small populations that may find it hard to attract attention and support.
			B.4.2. Social Values	B.4.2.1. Challenge: How to Address Social Values and Expectations	Social value is not always well defined, and it can be difficult to allocate finite resources to marginalized people or rare diseases since they may not benefit the majority – which can make it difficult for broader acceptance of decisions / outcomes.
				B.4.2.2. Conclusion: Importance of Social and Patient Values	The values of society and patients themselves are sometimes considered as an additional decision-making criterion but may be difficult to quantify.
				B.4.2.3. Conclusion: Using Tools to Measure Social Benefit	The use of tools to quantify and measure social benefits can be particularly helpful when attempting to incorporate social values or the impact of new therapeutics or interventions on broader society.
		B.4.B. The Importance of Patient Voice: Patients and their families (especially for paediatric populations) play an increasingly critical role in providing data, supporting analysis and reviewing healthcare funding decisions; using patient group representatives can be an effective approach to patient engagement and advocacy.	B.4.3. Societal Expectations	B.4.3.1. Conclusion: Governments Influence Social Adoption	Government institutions and regulatory authorities not only play an important role in introducing new drugs or treatments but many explicitly address social inequities by promoting social healthy policies; politics play a similar role.
				B.4.3.2. Conclusion:	Do not forget the effect of rare or chronic disease on caregivers; caregiver burden appears to be becoming a more and more important

Category	Subject	Theme	Topic	Conclusion or Challenge	Description
				Importance of Caregivers	consideration in evaluating disease treatment options.
				B.4.3.3. Conclusion: Society has Varied Expectations	Perhaps as expected, society and broader public stakeholders have a wide variety of perspectives on healthcare decision-making - some stressing the importance of equity while others focused on broader economic and / or environmental considerations.
				B.4.3.4. Conclusion: Society Still Values Patient Preferences	Although society may place importance on other criteria beyond the patient impact, many studies still place substantial importance on patient preferences when it comes to drug or therapeutic treatment evaluation.
C. Procedural Considerations	C.1. Stakeholder Considerations	C.1.A. The Importance of Expertise: Having appropriate subject matter expertise, specifically clinical as well as procedural, is critical to making well-informed funding decisions, especially when robust data or evidence is lacking in areas such as rare or orphan drugs.	C.1.1. Authority	C.1.1.1. Conclusion: Importance of Process Experts	Effective and rigorous decision-making processes require people who understand HTA or related MCDA in healthcare processes and can guide stakeholders through the process.
				C.1.1.2. Conclusion: Importance of Subject Matter Expert Representation	Having experts with subject matter knowledge is important in decision-making to both enable trust by stakeholders in the process, as well as to enable consensus making, as well as provide information when supporting evidence is lacking.
			C.1.2. Representation	C.1.2.1. Challenge: Clinicians as Advocates vs Experts vs Resource Stewards	There may be ethical conflicts as clinicians play multiple roles: advocate for their patients vs act as clinical subject matter experts vs act as responsible healthcare resource stewards.
		C.1.B. The Importance of Diverse Stakeholder Representation: Incorporating a wide variety of decision-making stakeholders, including patient and public representatives, not only		C.1.2.2. Challenge: Decision-making Membership -	Having long-term or permanent decision-makers can promote consistency and improve efficiency of decision-making processes but is also subject to potential bias / conflicts of interest.

Category	Subject	Theme	Topic	Conclusion or Challenge	Description
		helps promote a fulsome discussion and provides important real-world evidence, but also helps promote accessibility to those with less means to participate in the process.		Permanent vs Term-Limited	
				C.1.2.3. Challenge: How to Quantify Patient and Public Input	If decision-makers believe patient and public input are important, what is the best way to quantify that input or present such input with a similar sense of assessment of the quality of such input?
				C.1.2.4. Challenge: Patients May Have Vested Interests	Care must be taken when engaging patient groups as they may have vested interests in the procurement of certain treatments.
		C.1.C. The Increasing Importance of Public and Patient Input: Representatives from patient groups and the general public are becoming commonplace to ensure that funding decisions are made with the end patient in mind but also not to omit the impact that funding decisions have on other citizens e.g., broader healthcare system effects and limited funding of other public interest areas, whether health-related or otherwise.		C.1.2.5. Challenge: Public Stakeholders May Have Subconscious Biases	Different groups of public representatives / citizen groups may have their own biases.
				C.1.2.6. Conclusion: Identification of Key Stakeholder Representatives	Key stakeholders that are seemingly universally included in decision-making include healthcare practitioners, academics, managers, government bodies or oversight committees, patients and the general public.
				C.1.2.7. Conclusion: Importance of Engaging Patient Groups	Engaging patient groups in decision-making requires careful consideration including their role, their ability to participate, their interests, and level of process understanding.
		C.1.D. The Challenge of Vested Interests: It can be difficult to balance differing viewpoints as well as different values that stakeholders place on health and health interventions from an overall process perspective as well as difficult for some		C.1.2.8. Conclusion: Importance of Patient Input	The patient perspective is important not only for inclusivity and ultimate buy-in, but also because they can be a valuable source of information especially in rare diseases.
				C.1.2.9. Conclusion:	Input from the public as stakeholders is important and promotes social equity as well as transparency

Category	Subject	Theme	Topic	Conclusion or Challenge	Description
		stakeholders to balance their own competing priorities.		Importance of Public Input	and ultimate public buy-in. This is particularly important for publicly funded institutions.
				C.1.2.10. Conclusion: Importance of Stakeholder Accessibility	Efforts may be required to reach specific stakeholder groups e.g. lower socioeconomic status to promote social equity.
				C.1.2.11. Conclusion: Importance of Breadth of Stakeholder Input	Almost all decision-making processes involved stakeholder representation from wide variety of interests.
	C.2. Process and Timing Considerations	C.2.A. The Importance of Consistency: A documented consistent process used to support funding decisions not only complements the notions of transparency and accountability, but also helps avoid biases (e.g. recency bias) or undue influence from stakeholders as well as helps justify difficult or unpopular decisions.	C.2.1. Addressing Uncertainty	C.2.1.1. Conclusion: Importance of Addressing Uncertainty	Regardless of source of data and processes used for decision-making, it is important to acknowledge and / or address uncertainty in the data and process to promote transparency.
			C.2.2. Consistency and Precedence	C.2.2.1. Challenge: How to Balance Consistency across Different Patient illnesses and Interests	Consistency may be difficult to achieve while recognizing the inherent differences among illness types and patient interests.
				C.2.2.2. Challenge: Transparency vs Consistency	Which is more important - transparency or consistency of process? Is a consistent process more transparent?
				C.2.2.3. Conclusion: Rigorous Evaluation of Requests	A rigorous and transparent process will include a process or tool to filter out inappropriate requests for review.

Category	Subject	Theme	Topic	Conclusion or Challenge	Description
		C.2.B. The Importance of People and Timeline Clarity: Having clearly defined decision-making timelines and stakeholder roles helps avoid unnecessary delays in funding decisions and also helps all stakeholders understand theirs and others' expectations in the process, especially when decisions are taken at different levels of the healthcare system (e.g. hospital level vs national level).		C.2.2.4. Conclusion: Importance of Role Identification for Stakeholders	Whether using adaptive processes or consistent processes, it is important to ensure stakeholders know their role in the overall process.
				C.2.2.5. Conclusion: Inconsistent Results OK if Transparent Process	The results of decision-making do NOT need to be consistent over time as long as the process, criteria and evaluation process is transparent.
				C.2.2.6. Conclusion: Importance of Meaningful Supporting Data	Data and information used to support individual criteria should be clear, comprehensive, feasible to collect and understandable to stakeholders to enable effective decision-making.
				C.2.2.7. Conclusion: Processes Should be Documented	Decision-making processes should be documented to promote consistency and transparency.
		C.2.C. The Challenge of Consistency: A consistent funding decision-making process can be difficult to achieve, especially if having to balance competing priorities from different stakeholders, changing political climates, or when new evidence contradicts previous evidence or decisions.		C.2.2.8. Conclusion: Consistency Promotes Trust	The consistency of the process used for decision-making allows for increased transparency and builds trust among stakeholders.
			C.2.3. Timing and Frequency	C.2.3.1. Conclusion: No Timeline Specification	Several articles do not even mention the specific timeline within which decisions were made, perhaps suggesting that it authors ignore the importance of making timely decisions or the cost / opportunity cost of decision-maker and other stakeholders' time.
				C.2.3.2. Conclusion: Timelines for Decision-making Vary Greatly	The timelines for decision-making vary by scope and size of request; larger reviews or national health technology assessments (HTAs) often take months while small institutional requests can take days.

Category	Subject	Theme	Topic	Conclusion or Challenge	Description
				C.2.3.3. Conclusion: Timeliness of Decision-making	Having timely data collection and analysis enables higher stakeholder buy-in and reduces cost / time delays / anxiety associated with longer decision times.
	C.3. Communication Considerations	C.3.A. The Importance of Customizing Communications: Customizing communications for different audiences is important; it helps make information and decisions accessible, relatable especially if provided within a context to which local stakeholders can appreciate, and understandable to a wide variety of stakeholders if provided in lay or easy to understand language.	C.3.1. Messaging Design	C.3.1.1. Conclusion: Customizing the Communication of Results	Results may need to be customized to different stakeholders as different audiences will have different areas of focus.
				C.3.1.2. Conclusion: Importance of Local Context	Provide local / relevant context for decision-makers so that they understand the relevance of information to them.
		C.3.B. The Importance of Transparency: Transparency is paramount in both how the decision-making process occurs as well as what and how the decision was achieved; it enables buy-in and understanding from stakeholders as well as helps identify and avoid potential conflicts of interest that may occur among decision-makers or other stakeholders.		C.3.1.3. Conclusion: Importance of Plain Language	Provide information in plain language or understandable language for lay decision-makers.
				C.3.1.4. Conclusion: Synthesis of Decision-making Information	Synthesize information and data to help decision-makers provide input and informed decisions in a timely manner.
		C.3.C. Barriers to Transparency: Transparency can be difficult to achieve with barriers such as tight timelines, difficult to understand medical or scientific concepts, proprietary information, and language or cultural variability causing challenges for decision-makers to overcome.	C.3.2. Transparency	C.3.2.1. Conclusion: Avoid Conflicts of Interest	Explicitly avoid conflicts of interest to promote transparency and public trust.
				C.3.2.2. Conclusion: Clear Definitions	Clearly define key terms, inclusion / exclusion criteria, and any other important concepts to avoid confusion.
				C.3.2.3. Conclusion: Sensitivity Trumps Transparency	Sensitive information may be withheld from public (e.g. financial information, personal / private health info).

Category	Subject	Theme	Topic	Conclusion or Challenge	Description
				C.3.2.4. Challenge: Transparency is Difficult	Transparency can be difficult to achieve given different levels of education of stakeholders, complexity of information (especially scientific), and often variable timelines of process.
				C.3.2.5. Conclusion: Transparency Levels Vary	Transparency varies significantly in decision-making, especially for health technology assessments (HTAs), as HTA processes can differ dramatically by institution / location.
				C.3.2.6. Conclusion: Importance of Transparency of Process	Provide information on what and when is the decision-making process is important for enabling stakeholder input and understanding.
				C.3.2.7. Conclusion: Importance of Transparency of Results	Publicly sharing the results of the decision is an important element of overall transparency.
	C.4. Evaluation and Appeals Considerations	C.4.A. The Importance of Post-Decision Monitoring and Evaluation: Continued monitoring for real-world evidence and evolution of scientific data after funding decisions have been made helps decision-makers track the effectiveness of prior decisions and also enables stakeholders to appeal and / or improve upon previous decisions in light of new data or evidence.	C.4.1. Appeals Process	C.4.1.1. Conclusion: Value of Formal Appeals Processes	A formal appeals process that can be accessed by public and other stakeholders helps ensure appropriateness of past decisions and ensures transparency and consistency in the review process.
			C.4.2. Post-Decision Evaluation	C.4.2.1. Conclusion: Importance of Post-Decision Evaluation	Periodic review of prior evaluations should consider real-world evidence (RWE) to ensure ongoing relevance and effectiveness of decisions in light of changing information.
	C.5. Data Collection and Storage Considerations	C.5.A. The Value of Systematic Data Collection and Storage: Healthcare funding decisions are not always efficient but most established national and international decision-making bodies have incorporated the use of data templates and databases that allow for comprehensive, systematic, easy to	C.5.1. Data Collection	C.5.1.1. Conclusion: Value of Anonymous Voting	Use anonymous voting techniques to avoid peer pressure and ensure equal opportunity to voice opinions.
				C.5.1.2. Conclusion:	Data collected should be validated to ensure objectivity.

Category	Subject	Theme	Topic	Conclusion or Challenge	Description
		use and secure data collection and storage, and enable the opportunity for anonymous participation from stakeholders to embolden those who may feel disempowered to voice their opinions or provide information.		Importance of Data Collection Validation	
			C.5.2. Data Storage	C.5.2.1. Conclusion: Advantages of Databases	Databases provide information and data reliability and security for decision-making.
			C.5.3. Data Templates	C.5.3.1. Conclusion: Value of Submission Templates	Data or submission templates can help make the process more efficient, improve quality and encourage stakeholder participation, especially for patients.
			C.5.4. Tools / Decision Aids	C.5.4.1. Conclusion: Easy to Use Tools	Tools should be simple and easy to operate to facilitate effectiveness and timeliness.
D. Guiding Principles and Frameworks	D.1. Decision-making Frameworks	D.1.A. MCDA as the Default Decision Aid: Healthcare funding decision-making is increasingly using established economic tools or decision-making tools such as Multiple Criteria Decision Analysis (MCDA), which has formed the backbone for many healthcare technology assessments (HTAs) around the world.	D.1.1. Multiple Criteria Decision Analysis	D.1.1.1. Conclusion: Popularity of MCDA	Multiple Criteria Decision Analysis (MCDA) is the most commonly used decision aid for healthcare decision-makers but variants and other decision tools are increasingly being used.
			D.1.2. National Frameworks	D.1.2.1. Conclusion: Incorporation of MCDA into National Decision-making Frameworks	Many national and international institutions have incorporated multiple criteria decision analysis (MCDA) into their own documented process for healthcare funding decision-making.
	D.2. Ethical Frameworks	D.2.A. Utilitarianism Forms the Foundation of Economic Analysis: A utilitarian philosophy has traditionally been the foundation for healthcare funding decisions, hence the use of economic models in most established institutional funding processes.	D.2.1. Common Frameworks	D.2.1.1. Conclusion: Predominance of Utilitarian Approach	The majority of decision-making approaches incorporate a utilitarian view to justify their decisions; in particular looking at first the health effect, then economic effect and more recently the broader societal effect of interventions and alternatives.

Category	Subject	Theme	Topic	Conclusion or Challenge	Description
		D.2.B. The Need for a Transparent Ethical Framework: The use of a recognized transparent ethical framework such as the Accountability for Reasonableness (A4R) is gaining in popularity.		D.2.1.2. Conclusion: Importance of Accountability for Reasonableness	Many authors argue for the importance of using a transparent ethical framework throughout the decision-making process, of which the A4R framework is often cited.
	D.3. Quality Data Frameworks	D.3.A. The Need for a Recognized Evidence Evaluation System: Using a recognized system to evaluate the quality of scientific evidence helps achieve common understanding between stakeholders.	D.3.1. Evidence Frameworks	D.3.1.1. Conclusion: Prevalence of GRADE	Many decision-makers use the process of rating the quality of the best available evidence and developing health care recommendations following the approach proposed by the Grading of Recommendations, Assessment, Development and Evaluation (GRADE) Working Group.
	D.4. Other Ethical Considerations	D.4.A. Conflicting Ethical Principles: Authors and institutions are increasingly placing more importance in the justice domain of healthcare ethics by paying particular attention to special populations, accessibility / affordability, and the rule of rescue.	D.4.1. Application of Ethical Principles	D.4.1.1. Conclusion: Variety of Ethical Principles	Many different ethical principles are used in healthcare decision-making and HTAs in particular are increasingly moving beyond the four commonly cited principles in medicine (autonomy, justice, beneficence and non-maleficence) to incorporate important but often conflicting concepts such as the Rule of Rescue, Accessibility / Affordability, and Needs-Solidarity.
E. Operational Activities	E.1. Implementation Considerations	E.1.A. The Importance of Feasibility: Effective healthcare decision-making processes explicitly acknowledge the importance of a practical process as well as the feasibility of implementing the funding decision.	E.1.1. Implementation Feasibility	E.1.1.1. Conclusion: Feasibility Increasingly Important	Many institutions are focusing on the feasibility of decision-making - including the feasibility of collecting data, feasibility of implementing the decision, as well as specific treatment feasibility considerations including feasibility of the provision of treatment and even the feasibility of diagnosing the illness especially if it is a rare disease.
		E.1.B. Internal Operations Focus: Some institutions keep operational aspects of implementing a funding decision including supply chain reliability and internal accounting practices as factors that influence their healthcare funding decisions.	E.1.2. Supply Chain	E.1.2.1. Conclusion: Importance of Supply Chain Reliability	Some decision-makers, mostly at the national level, place a priority on brand name or drugs that have a reliable and high quality supply chain. Perhaps this becomes an even more important consideration during a global pandemic as some materials and supplies are becoming in short supply.
			E.1.3. Internal Accounting	E.1.3.1. Conclusion: Separate Pools of	Dedicating a separate pool of funds for specific diseases or institutional purposes has benefits and drawbacks.

Category	Subject	Theme	Topic	Conclusion or Challenge	Description
				Funds Have Pros and Cons	
F. Paediatric / Rare Disease Conclusions	F.1. Quality Evidence	F.1.A. Lack of Academic Literature: Based on our review of the academic literature, there may be an increasing but still relatively small percentage of publications describing funding approaches to rare / orphan drugs decision-making; there is a shortage of such literature focused on special populations such as paediatrics.			
	F.2. Limited High Quality Data	F.2.A. Shortage of Quality Evidence: High quality evidence such as Randomized Control Trials (RCTs) for rare diseases and paediatric populations are lacking due to limited patient populations and / or ethical constraints (in paediatric populations in particular), which makes it difficult to determine the comparative benefits of new interventions.			
		F.2.B. Uncertainty / Importance of Real-World Evidence: Given the lack of high quality data and significant amounts of uncertainty in novel treatments for rare / orphan diseases and in some paediatric populations, the importance of incorporating real-world evidence (RWE) becomes even more important.			
		F.2.C. Importance of Transparency: Institutions are likely to give greater weight to the transparency of process and results when there is a lack of high quality evidence to support funding decisions.			
	F.3. Specific Ethical Challenges	F.3.A. Difficulty of Justifying Resource Allocation: There is an inherent ethical challenge for rare / orphan drugs and paediatric populations where the utilitarian view of resource allocation does not necessarily support the high cost of drugs and / or relatively small populations associated with rare / orphan drugs and children compared to common illnesses and adult populations.			
		F.3.B. Importance of Indirect Financial Impacts: The indirect economic impacts of funding decisions on stakeholders such as caregivers of paediatric patients and children of patients of rare / orphan diseases seems to be given higher significance in decision-making by several institutions.			
		F.3.C. Different Values for Special Populations: While institutional stakeholders seem to want to prioritize special populations or patients with rare / orphan diseases, the broader public or stakeholders often do not share that viewpoint, making it more difficult to justify spending decisions that are funded by public dollars.			
